# Moving Beyond Moderation to Identify Differential Treatment Effects of Cognitive Behavioural Therapy for Childhood Anxiety: A Systematic Review

**DOI:** 10.1007/s10567-026-00564-8

**Published:** 2026-03-06

**Authors:** Lizél-Antoinette Bertie, Andrew Mackinnon, Emma A. McDermott, Maddison O’Gradey-Lee, Katarina Kikas, Mark Tomlinson, Jennifer L. Hudson

**Affiliations:** 1 School of Psychology, University of New South Wales, Sydney, Australia; 2 Black Dog Institute, University of New South Wales, Hospital Road, Randwick, Sydney, NSW 2063 Australia; 3 Department of Global Health, Faculty of Medicine and Health Sciences, Institute for Life Course Health Research, Stellenbosch University, Cape Town, South Africa; 4 School of Nursing and Midwifery, Queens University, Belfast, UK

**Keywords:** Childhood anxiety, Cognitive behavioural therapy (CBT), Interaction effects, Subgroup analyses, Moderator analyses, Differential treatment effects

## Abstract

Objective: Despite growing momentum towards personalised mental health care for children and adolescents, a central question remains unresolved: *which treatment works best for which child, and under what circumstances?* Identifying moderators of differential treatment effects is key to answering this question and guiding clinicians in tailoring interventions for childhood anxiety. However, progress has been hindered by conceptual ambiguity, inconsistent reporting, and methodological limitations in how moderators are defined, tested, and interpreted. This systematic review synthesises research on moderators of cognitive behavioural therapy (CBT) for childhood anxiety, aiming to determine whether any moderators are sufficiently consistent and robust to inform clinical decision-making. Method: A systematic search of the literature delivered records from inception to July 2025. Randomised controlled trials of CBT for youth with anxiety disorders (5—18 years) were considered, including studies that reported explicit statistical moderator interactions. Results: Of the 355 full-text articles reviewed, 68 studies originating from 44 randomised controlled trials met the inclusion criteria, reporting 28 significant moderators. Seventeen moderators showed a singular instance of differential treatment effect. The remaining 11 moderators showed multiple instances and were examined in greater detail. Although five moderators showed some evidence for differential treatment effects (age, Social Anxiety diagnosis, baseline anxiety severity, parent psychopathology, and parent acculturation), we cannot conclude that any reliable moderators of childhood anxiety outcome have been identified. Conclusion: This review represents a critical step forward in our search for factors that can guide optimal treatment selection in practice. Although these findings require confirmation through prospective studies, they offer a valuable foundation for precision mental health care.

**Supplementary Information:**

The online version contains supplementary material available at https://doi.org/10.1007/s10567-026-00564-8.

## Introduction

Evidence-based interventions for child and adolescent anxiety disorders, particularly cognitive behavioural therapy (CBT), are characterised by substantial heterogeneity in treatment outcomes. Although CBT is the best-established treatment for anxiety disorders (Sigurvinsdottir et al., 2020) only around half of children and adolescents respond positively following intervention (James et al., 2020). For the remaining half, further divergence is demonstrated through varying treatment response patterns during the follow-up period (Bertie et al., 2024a, 2024b).

This variability has prompted extensive investigation into the heterogeneity of treatment effects (Kessler & Luedtke, 2021), a framework that seeks to identify variables associated with treatment response. Within this framework, two types of variables are commonly examined: predictors, which estimate the overall likelihood of improvement regardless of treatment condition, and moderators, which identify for whom a particular treatment is more or less effective (Varadhan & Seeger, 2013). For example, age may predict overall treatment success regardless of the intervention received (predictor), but if older children receive more additional benefit from CBT versus treatment as usual than younger children, age functions as a moderator. Importantly, moderators are not simply correlates of outcome; they reflect differential treatment response, indicating that the effect of treatment varies across subgroups. Understanding moderation in this way is essential for advancing personalised care, where treatment decisions are tailored to individual characteristics.

### Current Evidence

Although there is reliable evidence for predictors of CBT outcomes for childhood anxiety disorders (Kunas et al., 2021), evidence for moderators of CBT response is limited and inconsistent (Nilsen et al., 2013; Norris & Kendall, 2020; Zhou et al., 2025). Yet, moderators are the only variables capable of identifying which children are likely to benefit more from CBT compared to alternative treatments (Kraemer, 2016). Previous reviews have struggled to yield clinically actionable insights. Nilsen et al. (2013) and Norris and Kendall (2020) noted that most studies focused on demographic and clinical variables of convenience (e.g., age, sex, baseline severity), often without a strong theoretical rationale and with inconsistent results. More recently, Zhou et al. (2025) conducted a meta-analysis examining sample and intervention characteristics as moderators across both prevention and treatment trials, but their scope was limited to standardised CBT protocols compared to non-active controls, excluding individually tailored or disorder-specific interventions. The current review addresses these limitations by applying a structured framework to enhance the replicability and interpretability of clinically relevant moderators. It extends prior work by integrating findings across a broader range of study designs and theoretical models, including both standardised and personalised CBT approaches, comparisons with active controls, and emerging analytic methods. This approach aims to clarify conceptual and methodological gaps, highlight underexplored moderators (e.g., parental accommodation, reward sensitivity), and propose a roadmap for advancing personalised intervention strategies for childhood anxiety treatment.

### Conceptual and Methodological Mismatches in Moderator Research

The absence of definitive moderators, that is, moderator variables that are universally and consistently identified across studies, is not unique to the field of childhood anxiety. It reflects a broader challenge in youth psychotherapy, often referred to as the aspiration-method mismatch theory (Mullarkey & Schleider, 2021). This theory describes the fundamental research gap between our aspirations for empirical clarity and the methods we use to achieve those aspirations. Mismatches partially explain how methodological limitations and conceptual misunderstandings have hindered progress in identifying meaningful moderators of treatment heterogeneity. For example, key issues include the common practice of testing *one moderator at a time*, which reduces statistical power and increases the likelihood of false negatives; *limited sample sizes*, which constrain the detection of small but clinically meaningful effects; and *group-level designs*, which often fail to generalise to individual-level treatment decisions.

Although some of these challenges have been acknowledged in reviews of personalisation in youth mental health (e.g., Bertie & Hudson, 2021), additional challenges contribute to the lack of evidence on actionable moderators. One challenge is the **language** used in moderator research, which varies considerably and is often confusing for clinical audiences. Terms such as differential treatment effect, heterogeneity of treatment effects, moderation effect, effect modification, interactions, subgroup comparisons, or moderator analysis are frequently used interchangeably or incorrectly (Hancock & Kent, 2022). Clarifying these concepts is essential, as they carry distinct theoretical and statistical implications that affect interpretation and application.

A second challenge involves misconceptions about the **nature and timing of moderator variables** within RCT designs. Following Kraemer (2016), variables measured before randomisation are considered *baseline factors*, while those measured after randomisation are *intervening factors*. Only baseline factors can function as moderators, interacting with treatment conditions to influence outcomes. Intervening factors, while potentially informative, cannot indicate differential treatment response and may instead reflect mechanisms of change or mediators (Wang & Ware, 2013). Additionally, whether a variable is modifiable or fixed influences its interpretability. Time-varying factors measured as baseline (i.e., comorbid diagnoses) may be influenced by the intervention or confounded by other variables, complicating their role and potentially indicating more complex models such as moderated mediation (Kraemer et al., 2006).

A third challenge involves the **variability in study design**, particularly in child anxiety research. Differences in assessment practices (diagnostic vs. symptom measures), the timing of the assessment (post-treatment vs. follow-ups), informant sources (i.e., parent, child, or clinician report), outcome definitions (remission, response, functional improvement), and the type of outcome measure (binary vs. continuous) all significantly influence the detection and interpretation of differential treatment effects (Brankovic et al., 2019; Wang & Ware, 2013).

A fourth challenge lies in understanding **how moderators are assessed** in psychotherapeutic research. Moderator analyses typically examine whether the effect of a treatment *T* on an outcome *Y* varies based on a third variable *M*–the moderator (Bansak, 2020). When this occurs, a *statistical interaction* is said to be present (Brankovic et al., 2019). A common approach to testing moderation is stratification, also known as subgroup analysis, where participants are grouped based on their levels of the moderator variable (strata). Researchers must decide whether they are interested in (a) *within-subgroup effects*: estimating treatment effects separately for each subgroup (e.g., boys vs. girls), or (b) *across-subgroup effects*: comparing treatment effects between subgroups to test for interaction (Groenwold & Dekkers, 2023). For example, in an RCT comparing CBT to supportive counselling, researchers may hypothesise that treatment effects on anxiety symptoms differ by sex and comorbid depression symptoms. In this scenario, *within-subgroup* analysis would estimate the treatment effect separately for boys and girls, or for children with and without comorbid depression. However, this does not test whether the treatment effects differ significantly between subgroups; it only assesses whether each subgroup benefits from treatment relative to a null effect (Kraemer et al., 2006). In contrast, *across-subgroup* analysis directly tests for interaction by comparing treatment effects between subgroups. This is typically assessed using an interaction model that includes the treatment, moderator, and their interaction term (*T* + *M* + *T* × *M*) (Brankovic et al., 2019).

Relatedly, *across-subgroup effects* can be interpreted in two ways. First, as an **effect modification**, where the treatment effect differs across levels of the moderator (Varadhan & Seeger, 2013). For instance, if CBT is more effective than supportive counselling for girls but not boys, sex modifies the treatment effect. The second is **causal interaction** (Groenwold & Dekkers, 2023), where the researchers may explore whether the moderator is modifiable and they can intervene on *M* to change the effect of *T* on *Y* (Bansak, 2020). For example, if CBT is less effective than supportive counselling for children with comorbid depression, addressing the comorbidity may enhance treatment response. However, causal interaction requires stronger assumptions and more complex statistical methods, and is rarely tested in practice (Bansak, 2020). Although both within- and across-subgroup analyses offer valuable insights, their interpretation depends on the clinical motivation and the nature of the moderator. These distinctions underscore the conceptual ambiguity and methodological complexity inherent in moderator research. A full discussion of effect modification, causal interaction, and confounding is beyond the scope of this review, but is briefly revisited in the future reporting standards section.

A final and critical challenge is the nature of the control conditions used in RCTs. Whether the comparator is **active** (e.g., other therapeutic intervention) or **inactive** (e.g., waitlist) significantly influences the comparability, replicability, and interpretability of differential treatment effects (Karlsson & Bergmark, 2015). Comparisons of an intervention with inactive controls estimate *absolute effects*, that is, whether a subgroup of patients benefits from the intervention compared to receiving no treatment. In contrast, comparisons with active controls estimate *relative effects*, showing whether a subgroup of patients benefits more from one treatment than another (Karlsson & Bergmark, 2015). This distinction is crucial when synthesising moderator findings across studies, because without comparable control conditions, it becomes difficult to assess the consistency and reliability with which moderator effects have been identified.

### This Review

In sum, the variability in how moderators are conceptualised, tested, and interpreted has limited the clinical utility of existing research. Despite these challenges, the search for moderators remains central to the goal of personalised treatment in childhood anxiety. This systematic review builds on prior work by offering a structured synthesis of moderator evidence for CBT to identify variables that consistently and robustly predict differential treatment response. By integrating findings across diverse study designs, outcome measures, and analytic approaches, this review seeks to clarify the current evidence base and provide a foundation for future research and clinical application.

## Method

### Protocol and Registration

This systematic review was registered with the International Prospective Register of Systematic Reviews (PROSPERO: registration number CRD42021245781). The review methods were pre-specified prior to commencement, and no significant deviations from the protocol occurred. The review was conducted in accordance with the Preferred Reporting Items for Systematic Reviews and Meta-Analyses (PRISMA) guidelines (Moher et al., 2015).

### Eligibility Criteria

Studies were eligible for inclusion based on the following PICOS inclusion criteria:

*Population (P):* children and adolescents aged 5–18 years with a clinical diagnosis of one or more of the following primary anxiety disorders: generalised anxiety disorder (GAD), separation anxiety disorder (SAD), social anxiety disorder (SoAD), specific phobia (SP), panic disorder (PD) and selective mutism (SM). A diagnostic assessment of anxiety disorders at baseline was made according to DSM-IV and DSM-5 criteria (American Psychiatric Association, 2013). Studies focusing on obsessive‐compulsive disorder or posttraumatic stress disorder were excluded, as these are classified separately in the DSM‐5. Studies involving populations with neurodevelopmental disorders or chronic medical conditions were also excluded. However, studies including participants with comorbid conditions such as autism spectrum disorders (ASD), mood disorders (e.g., depressive disorder), and behavioural disorders (e.g., attention/deficit hyperactivity disorder, ADHD), were included if anxiety was the primary target of treatment.

*Intervention (I):* Interventions explicitly testing cognitive and/or behavioural therapy (CBT), with a focus on its effects on anxiety symptoms and disorders. Eligible interventions included standard CBT as well as any augmented (e.g., pharmacological or psychological) or modified versions (i.e., changes in duration or number of sessions).

*Comparator (C):* Any control condition was permitted. Only RCTs were included, as they are considered the gold standard for evaluating intervention efficacy and effectiveness. In addition, the investigation of moderators and differential treatment effects requires comparison with a control group (Hancock & Kent, 2022).

*Outcome (O):* Studies were required to use anxiety-specific measures to assess outcomes, including remission (the presence or absence of diagnosis), response (symptomatic improvement), or functional outcomes (i.e., anxiety-related improvement in daily functioning). A minimum of two assessment time points was required, including baseline and at least one post-treatment assessment.

*Study design (S):* Only RCTs were eligible for inclusion and could be conducted in any setting. The final inclusion criterion was the mention of moderation testing. Only interactions involving treatment condition (i.e., true moderators of differential treatment effects) were included. Interactions between other variables (e.g., child age × parental anxiety) were excluded.

### Search Strategy and Study Selection

Electronic databases Ovid PsycINFO, Ovid MEDLINE, Ovid EMBASE, and CINAHL were systematically searched using tailored search strings designed to balance specificity and sensitivity (Siddaway et al., 2019). Full search strategies for each database are provided in the Supplementary Information (A). The search was limited to peer-reviewed journal articles published in English. Narrative reviews, systematic reviews, and meta-analyses were excluded, although reference lists of these articles were screened for potentially eligible studies. Initial searches were conducted on 23 October 2023 and updated on 12 September 2024 and 11 July 2025 using the same strategy.

All identified records were imported into COVIDENCE (Veritas Health Innovation, 2020), a web-based platform for managing systematic reviews. Duplicate records were automatically removed. Study selection followed a two-stage screening process. First, title and abstract screening was conducted by a single reviewer, who excluded studies that clearly did not meet the PICOS criteria. Excluded studies were validated by a second reviewer (100%). Second, full-text screening was conducted by two reviewers from a pool of five, who assessed each article against the final inclusion criterion related to moderation testing. Discrepancies were resolved through discussion.

### Data Extraction

A custom data extraction template was developed, aligned with the protocol for this systematic review. The first author conducted the initial data extraction and classification, which was subsequently verified by a second author.

Data were extracted across four domains:

i. *Original trial details*: Included study title, first author, publication year, sample size, mean age, treatment and control conditions, and treatment duration (measured in number of sessions).
ii. *Moderator study details*: Included the first author and publication year, grouped according to the original trial to which the moderator study was linked. Anxiety outcomes were indexed as: *S (Status)*: Binary diagnostic outcome (presence/absence of disorder); *R (Response)*: Continuous measure of anxiety symptomology; and *F (Functioning):* Additional measures related to functioning or comorbidity. Details were also extracted on the anxiety outcome instruments used, the reporting source (i.e., clinician, child, or parent report). The number and timing of assessment occasions were also extracted.
iii. *Moderators of differential treatment effects:* All reported moderators (both non-significant and significant) were recorded, along with the instruments used to measure them. Moderators were classified as either time-variant or invariant according to whether they could be altered by the trial.
iv. *Statistical interaction details:* Information was extracted on the method of analysis (e.g., stratification or interaction modelling) and the statistical techniques (e.g., regression, machine learning).

### Analytical Plan

#### Quality Assessment (Risk of Bias)

Based on a consensus coding process, two from a pool of three reviewers, independently assessed the methodological quality of the included RCTs (Higgins et al., 2019) using the Cochrane Risk of Bias tool (v2) (Sterne et al., 2019). The tool assesses potential bias across five domains: (1) randomisation process; (2) risk of deviation from planned intervention; (3) missing outcome data; (4) measurement of outcome data; and (5) reporting of results. Each domain was classified as “low risk”, “unclear/some risk”, or “high risk” of bias. Discrepancies were resolved through discussion. This review takes a descriptive and inclusive approach. Rather than selecting a ‘correct’ analysis method in Domain 2 (e.g., intention-to-treat [ITT] or per-protocol [PP]), it documents the analysis strategy and the stated or implied estimand used in each study and considers whether misalignment may introduce bias to this domain (Silverman et al., 2024).

#### Quality Assessment (Moderator evaluation)

Based on a consensus coding process, two reviewers independently assessed the methodological quality of studies reporting statistical interaction testing using the Pincus et al. (2011) checklist. This checklist includes five binary (yes/no) criteria: (1) A priori hypothesis: was the moderating effect assessed as part of a pre-specified hypothesis? For secondary analyses (post hoc), this question was augmented with: (1b) A priori power analysis: was a power analysis for moderation reported in the original trial? (2) Theoretical or empirical basis: was the moderator variable selected based on theory or prior evidence? (3) Timing: was the moderator measured prior to randomisation? (4) Measurement quality: Was the moderator assessed using a psychometrically valid instrument? (5) Explicit interaction test: was the moderation effect tested through a formal interaction between the moderator and treatment? Based on these criteria, evidence was classified into three categories: confirmatory evidence (yes to all factors), exploratory evidence (yes to factors 3 and 4), and insufficient evidence (any other combination).

#### Control Condition Categories

Two reviewers independently classified all moderator studies according to the type of control condition used (James et al., 2020; Mohr et al., 2014). This stratification enabled a more refined interpretation of differential treatment effects and their clinical relevance. Control conditions were grouped as follows:

i. *Inactive controls:* Included waitlist, no treatment, or Treatment as Usual (TAU) when TAU involves providing support (i.e., instructions to patients to obtain existing services outside of those provided by the trial authors).
ii. *Active non-CBT controls*: Included TAU when used as a standard or non-manualised treatment within the study over the same period, meaning the intervention contained active ingredients but no CBT elements. This category also included alternative evidence-based treatments that did not incorporate CBT.
iii. *Active CBT controls:* Included variations in CBT delivery (e.g., individual vs group formats), augmented CBT protocols (e.g., additional modules), modified conditions (e.g., bibliotherapy), or other combinations of CBT-based interventions.

#### Synthesis Methods and Reporting

Due to substantial heterogeneity in statistical methods, outcome measures, assessment timing, and effect size reporting, a formal meta-analysis was not feasible. Instead, findings were synthesised descriptively in accordance with the Synthesis Without Meta-analysis (SWiM) guidelines (Campbell et al., 2020). This approach included: conducting quality assessments for both RCTs and moderator analyses; grouping studies by control condition; and describing synthesis methods and reporting.

To facilitate the synthesis of diverse effect measures and support the visualisation of differential treatment effects, this review employed an Effect Direction Plot (Boon & Thomson, 2020). This visual tool supports a clearer understanding of the consistency and potential clinical relevance of moderator findings in three ways:

i. *Direction of effects:* the plot indicates whether comparative subgroups experienced greater improvement, no difference, or less improvement following treatment.
ii. *Pattern of interaction:* the plot indicates whether the effect was observed in one condition or subgroup only (simple interaction); whether opposite effects were observed across one condition based on moderator levels (opposing crossover); whether opposite effects were observed across different condition based on moderator levels (crossover interaction); or whether the same direction of effect was observed across conditions (consistent interaction).
iii. *Replication:* the plot helps to identify whether similar differential treatment patterns were observed across multiple trials using comparable treatment conditions.

Finally, in the absence of formal criteria for determining whether a moderator is consistent and robust, this review applied a set of logical, albeit conservative, standards to identify emerging trends. Specifically, a moderator was considered to show a consistent and robust differential treatment effect (DTE) if it met the following criteria: (i) *Replication:* DTEs were observed in at least two independent studies (trial datasets); (ii) *Control category***:** Studies were conducted within the same control condition (e.g., active CBT control); (iii) *Uniform direction of effect:* The same subgroup consistently showed superior outcomes across studies; (iv) *Outcome measures:* Studies used similar outcome indices (e.g., remission, symptom reduction, functional improvement); and (v) *Timing:* Outcomes were evaluated at aligned assessment points (e.g., post-treatment or 1-year follow-up).

## Results

Results have been presented according to the approaches described in the Method and the Analytical Plan sections.

**Fig. 1:**
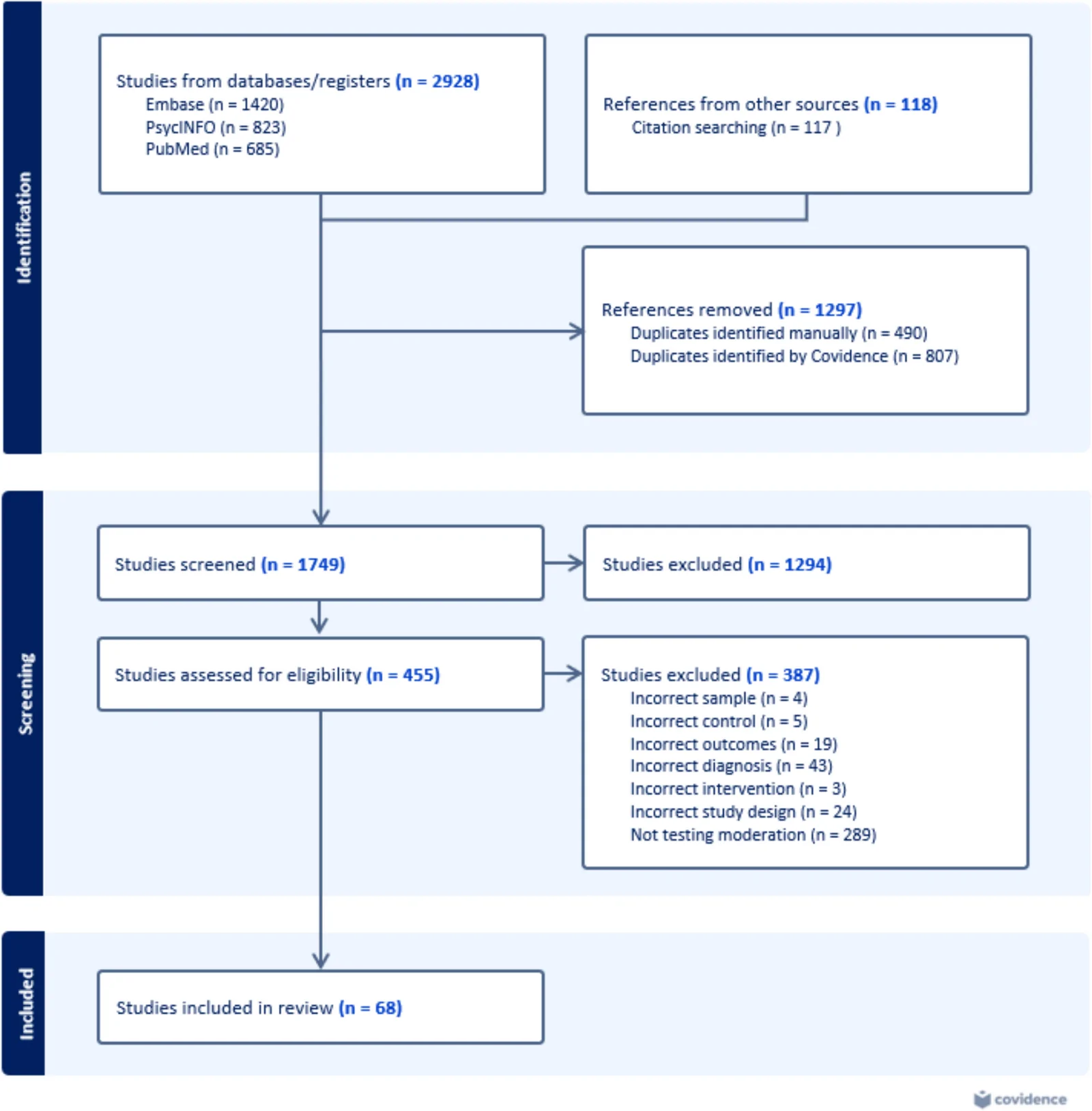
PRISMA flow chart of the study selection

*Study selection.* A PRISMA flow chart of the study selection is presented in Fig. 1. Our systematic search captured a total of 2928 articles, from which 1297 duplicates were removed. A manual search of reference lists yielded another 118 relevant articles. Following the screening of the resultant 1749 titles and abstracts, we removed 1294 irrelevant records. A total of 455 full texts were assessed for eligibility, and following the exclusion of 387 articles that did not meet the inclusion criteria, data were extracted for the remaining 68 articles in this systematic review. Further details are provided alongside the search strategy in the Supplementary Information (B).

*Moderator study characteristics.* Extracted moderator study characteristics, including sample and intervention information, are also presented in the Supplementary Information (C). The final 68 studies, published between 1996 and 2025, were based on 44 original trials, with sample sizes ranging from 31 to 488. Trial settings included research sites (i.e., university clinics, *n* = 30), a combination of private and community clinics (*n* = 9), home and school settings (*n* = 3), and primary care (*n* = 2). Assessment occasions ranged from two to five occasions (i.e., pre-, post-, and 3, 6, or 12-month follow-up measurements).

*Types of Statistical Interaction Tests.* Nearly all studies (*n* = 54, 98%) examined individual moderator variables, while one study examined a combined moderator (Wallace et al., 2017). Most studies (*n* = 52, 95%) used some form of conventional linear modelling, and three studies conducted more complex moderator investigations employing machine learning techniques (Lebowitz et al., 2021), a correspondence analysis technique (Kim et al., 2020a, 2020b), and a response surface analysis (Zilcha-Mano et al., 2021). The remaining studies that reported significant statistical interaction results were further categorised as interaction modelling (*n* = 22), stratification analyses (*n* = 6), and both (*n* = 3). From these studies, 62 variables were investigated as potential moderators, of which 23 predicted differential treatment effects based on significant interaction testing results, and the remaining 39 were inconclusive based on reports of insignificant results.

### Methodological Quality

#### Quality of Trials

**Fig. 2:**
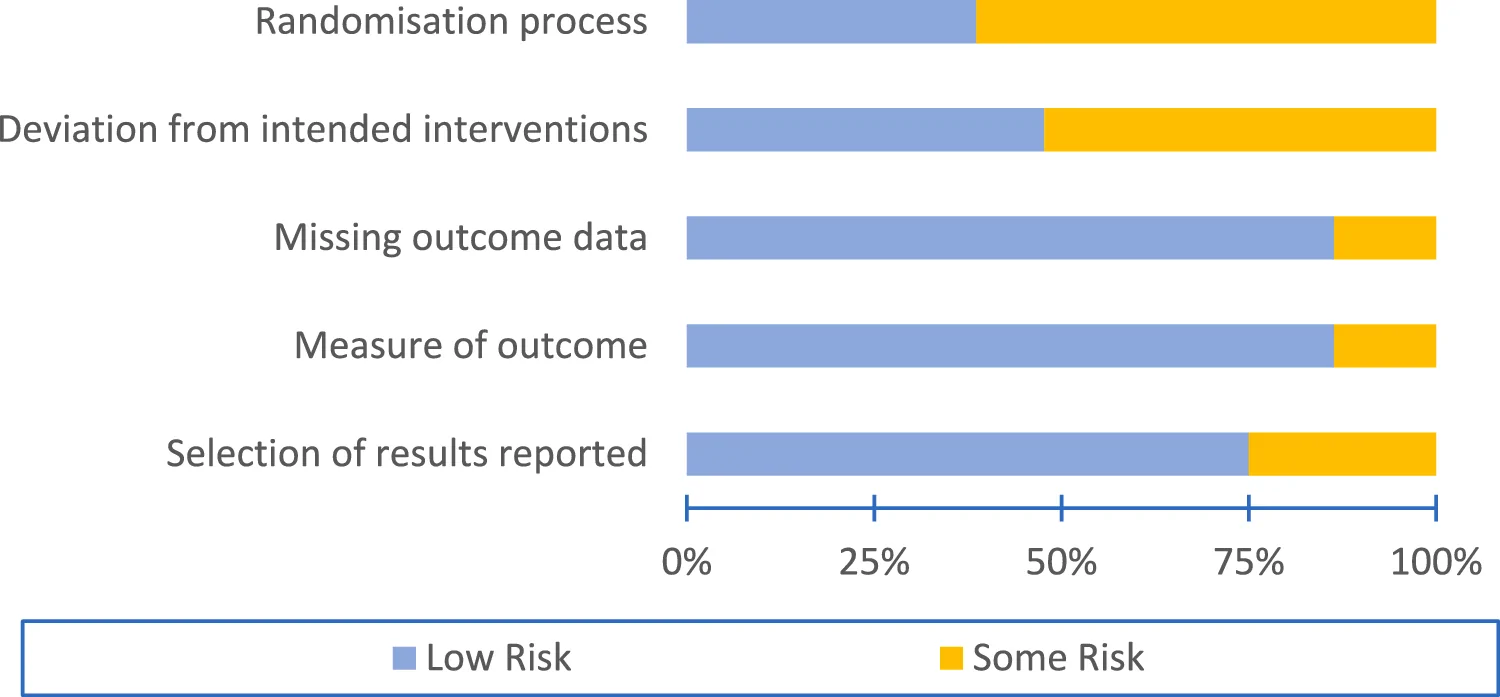
Risk of bias evaluation

Figure 2 presents a summary of the risk of bias assessment for the randomised controlled trials included in this systematic review. Detailed domain-specific analyses are available in the Supplementary Information (D).

*Risk of bias due to randomisation*: Of the 44 trials reviewed, 27 did not report sufficient information regarding the randomisation procedures or allocation concealment, indicating a potential risk of bias in this domain.

*Risk of bias due to deviations from intended interventions:* Blinding participants, families, and clinicians is often impractical in psychological trials for childhood anxiety (Zhou et al., 2025). In 40 of the 44 trials, intervention groups were known to all parties. Protocol adherence was reported as good for 36 trials, with 4 trials not providing any information on fidelity, integrity, or adherence. Regarding analysis strategies, 23 trials reported Intent-to-treat analysis (ITT) outcomes, 13 trials used per-protocol (PP) or completer analysis in contexts of low attrition, and 8 trials reported both. There was alignment between the chosen analysis strategy and the examined treatment effects in 24 trials (Tripepi et al., 2020). In the remaining 20 trials, misalignment informed the question of whether an appropriate analysis was conducted, resulting in a rating of some risk of bias.

*Risk of bias due to missing outcome data:* Of the 44 trials, 27 reported outcome data for most or all of their participants. Sensitivity analyses were conducted in 30 trials, with 20 providing information on missing patterns, and 26 trials reporting on attrition analyses, finding no difference between missing and non-missing samples. Of the remaining 17 trials, 6 found no differences between missing and non-missing samples and reported missing data patterns not associated with increased bias; 5 conducted sensitivity analyses but did not report missing data patterns, although they used methods that correct for bias; and 6 did not provide sufficient information on missing data, attrition analyses, or bias mitigation strategies.

*Risk of bias due to measurement of outcome:* Only 4 trials successfully blinded all groups during outcome assessments, and one trial had singular clinician-reported outcomes by a clinician blind to group allocation. The remaining trials showed some risk of bias because the outcomes (remission and response) were reported by clinicians, parents, and children, who were aware of group assignment, potentially influencing their responses.

*Risk of bias due to selection of the reported results:* Fifteen trials had preregistered protocols, and 17 trials included a data analysis plan that aligned with reported outcomes. The remaining 11 trials did not report a priori analysis plans but did report outcomes for all measures listed in the methods section at the specified time points. These were rated as having some, albeit small, risk of bias due to selective reporting.

#### Quality of Moderator Studies

**Fig. 3:**
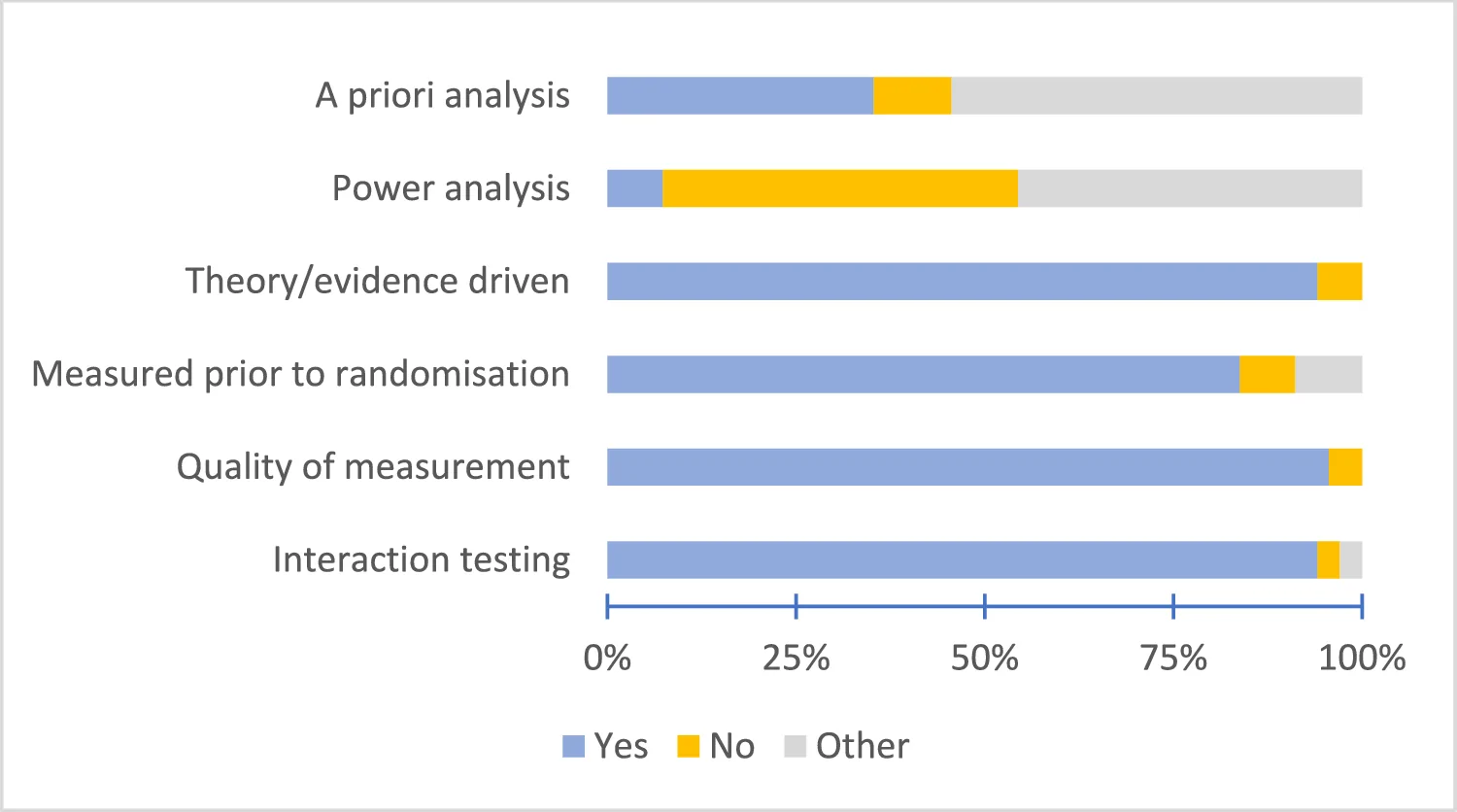
Moderator quality analysis

A summary of the moderator quality analysis is presented in Fig. 3. Among the 68 moderator studies evaluated, 38% (*n* = 26) were rated as having confirmatory quality, 40% (*n* = 27) achieved an exploratory quality score, 13% (*n* = 9) were deemed insufficient, and 9% (*n* = 6) were mixed insufficient when some variables were measured post-baseline. Details of the moderator quality assessment analyses are provided in the Supplementary Information (E).

Of the 31 original trial studies, 24 stated an a priori intention to examine potential moderators of treatment outcome (77%). The remaining studies were secondary analyses (*n* = 37), and only 14% (*n* = 5) included a power analysis or sample size estimate for assessing the ability to detect differential treatment effects. Most studies examined individual moderator variables that were justified in the text (*n* = 64; 94%), two studies did not offer specific information regarding their choice of moderator variables, and two studies used all available factors in statistical interaction testing (9%). Studies measured moderators before randomisation (84%, n = 57), with the remainder testing a moderator assessed after treatment commenced (*n* = 5), and a mix of pre- and post-baseline factors (*n* = 6). Moderators were measured using good-quality measures with three studies (6%) using novel or unvalidated measures not used before (i.e., single questions quantifying the moderator variable). Reassuringly, most studies employed statistical interaction testing in their moderation analyses (94%, *n* = 64), with only two studies using separate subgroup analysis, and two studies not providing enough information to determine the method used. All studies were retained for the systematic review to illustrate current practices and understanding in the field.

#### Control Condition Categories

**Fig. 4:**
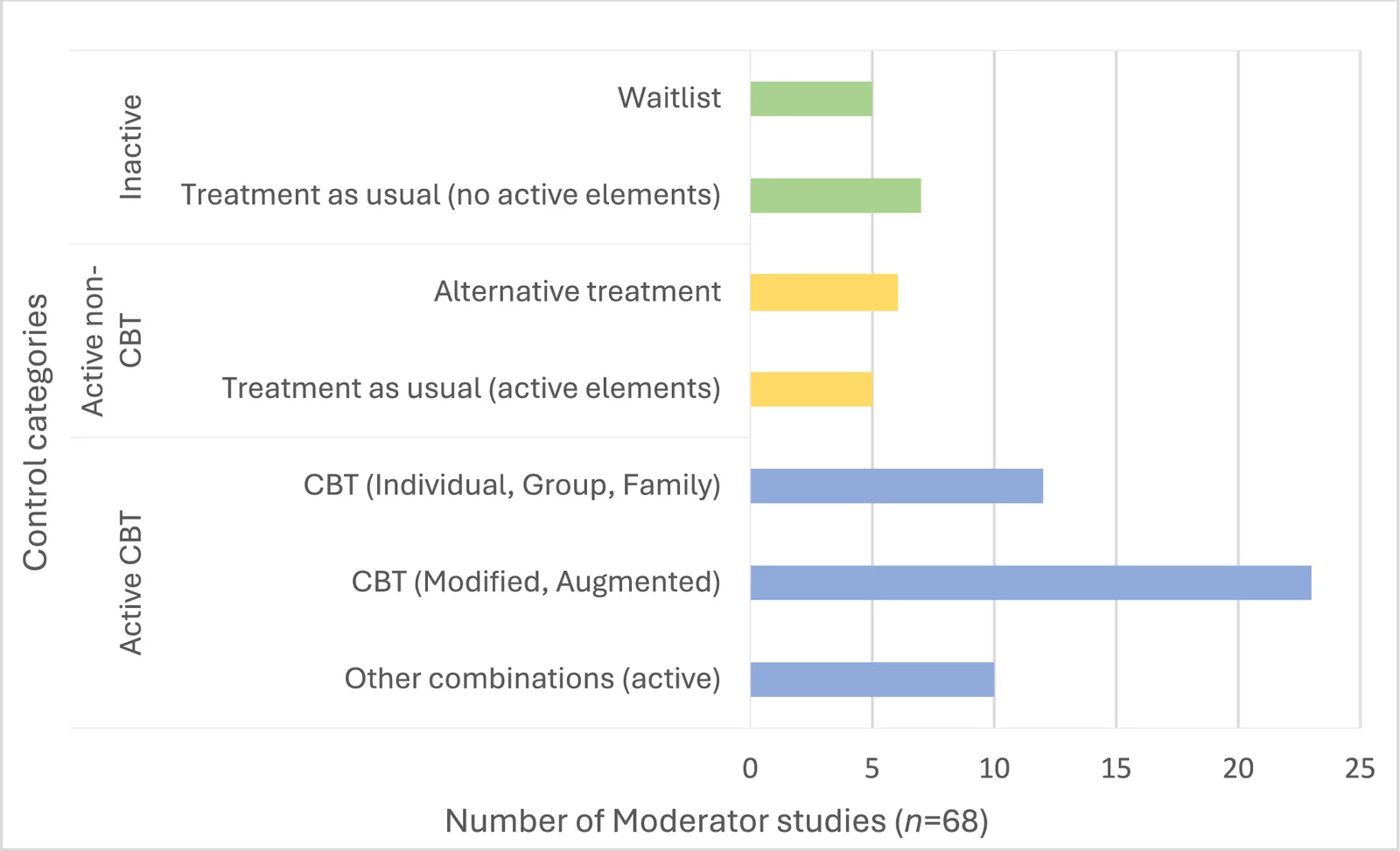
Control condition categorisation

Moderator studies (*n* = 68) were based on trials that compared any form of CBT with control conditions categorised as inactive (*n* = 12, 20%), active non-CBT (*n* = 11, 20%), and active CBT control conditions (*n* = 45, 60%). Figure 4 shows a summary of the categorisation of control conditions.

#### Moderators Associated with Differential Treatment Effects (DTE)

**Fig. 5:**
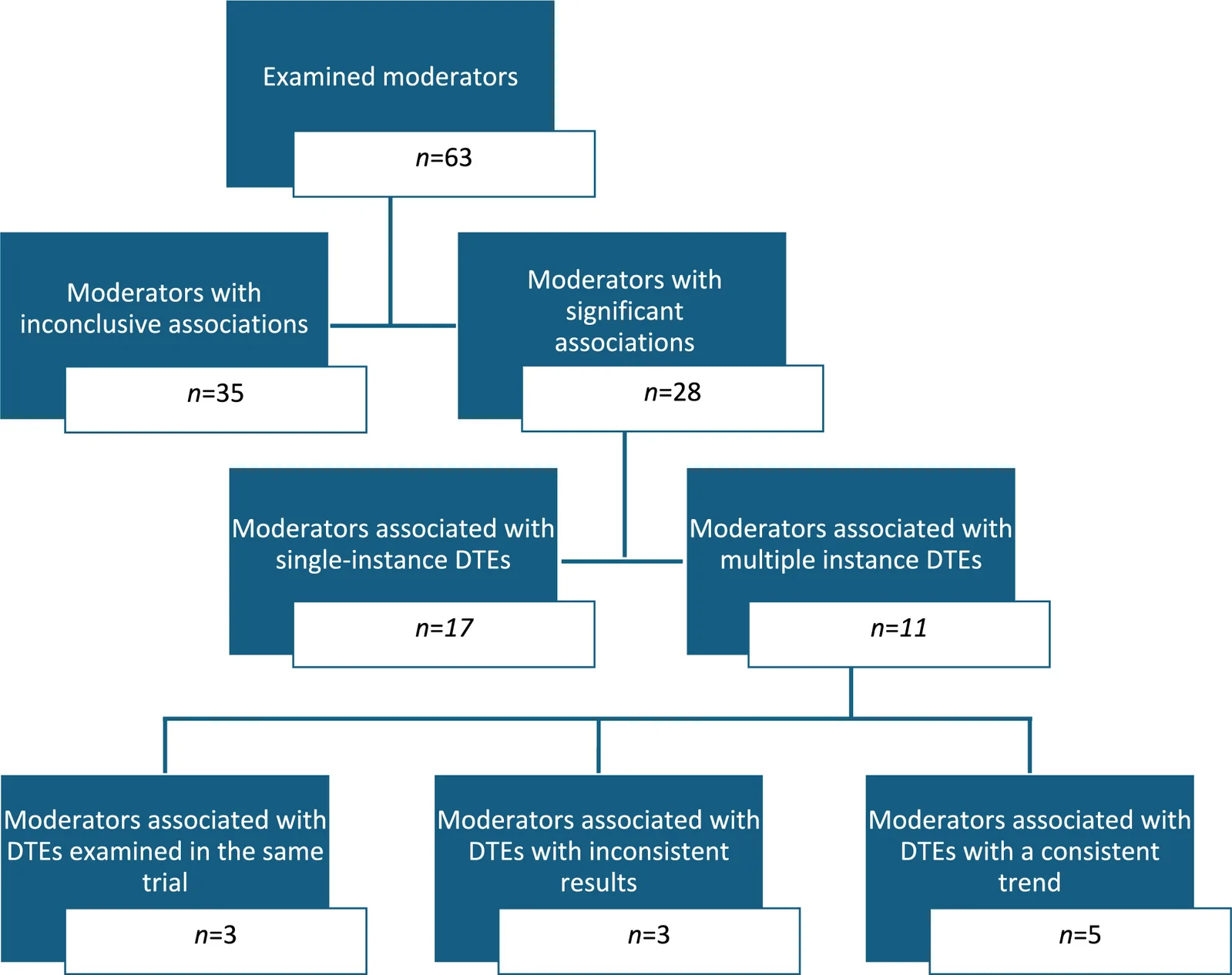
Moderator variables assessed in this systematic review

The flow of all examined moderators is presented in Fig. 5, and a detailed overview is provided in the Supplementary Information (F). Details of the 28 moderator variables that were significantly associated with differential treatment effects (DTEs) are presented in Table 1. Effect Direction Plots were generated for these 28 moderator variables that indicate which moderator variables had single instances of DTEs (i.e., observed only once) and which variables had two or more results across control conditions and are presented in Tables 2, 3, 4 by control condition category. Accordingly, the 11 moderator variables that demonstrated differential treatment effects in multiple studies are reported in greater detail below as either time-invariant or time-variant moderator variables.

**Table 1:** Moderator variables examined vs significantly associated with differential treatment effects

Variables	Type of variable	Number of times examined	Number of times significant	Single observations	Inactive controls	Active Non-CBT controls	Active CBT controls
*Demographic*							
1. Age	IV	32	7			2^†^	5^†^
2. Sex	IV	20	3				3^†^
3. Ethnicity	IV	9	4		3*		1
*Clinical*							
4. Primary diagnosis	V	14	6			1	5^†^
5. Baseline anxiety severity	V	9	4		1	3^†^	
6. Number of anxiety disorders		1	1	1			
7. Comorbid anxiety symptoms		3	3	3^			
8. Comorbid internalising/depressive symptoms	V	9	5	1^	2*	1	1
9. Comorbid externalising/ADHD symptoms	V	5	3	1^			2^†^
10. Comorbid autism symptoms	V	2	2				2^†^
11. Comorbid status (other diagnoses)		8	1	1			
*Contextual*							
12. Parent psychopathology	V	10	5				5^†^
13. Parental stress/negativity		2	1	1			
14. Family accommodation		3	1	1			
15. Parent acculturation	V	2	2				2^†^
16. Parent education		2	1	1			
17. Parental acceptance		2	1	1			
18. Parental reinforcement		2	1	1			
*Treatment*							
19. Therapeutic alliance		1	1	1			
20. Therapist adherence		3	1	1			
21. Therapist efficacy		1	1	1			
*Child/other*							
22. Coping ability		1	1	1			
23. Decision-making		1	1	1			
24. Child oxytocin	V	2	2			2*	
25. EMA sleep variables		1	1	1			
26. Interpersonal negative events		1	1	1			
27. Attention control		1	1	1			
28. Emotion regulation/dysregulation		2	1	1			

The type of variable is indicated by I (Invariant) and V (variant). ^Instances refer to individual comorbid subscale measures that were associated with DTEs on one occasion. *Moderators that have significant differential treatment effects based on the same trial dataset. ^†^Moderators that have more than one significant DTE in one control category for comparison

**Table 2:** Effect direction plot of moderator variables significantly associated with treatment outcome in inactive control conditions

Inactive control conditions												
CBT versus WL (None)												
CBT versus TAU												
Study author	Examined moderator	Measure	Index	Measure	Source	Timing of assessment	DTE	CBT	DTE	Control	Pattern	Interpretation of Improvement: greater improvement, no difference, or less improvement
Ollendick (2019)	Baseline anxiety	SCARED	R	SCARED	Child	3 m FU	▲	ABMT		ACT	C	Greater: lower baseline social anxiety (-1*SD*)
	Baseline anxiety	SCARED	R	SCARED	Child	3 m FU	▼	ABMT		ACT		Less: higher baseline social anxiety (+ 1*SD*)
	Attention control	EATQ-R	R	SCARED	Parent	Post	▲	ABMT		ACT	A	Greater: higher attention control
Weersing (2017)	Ethnicity	–	R	PARS	Clinician	Post	▲	BBT	▼	ARC	B	Greater: hispanic versus non-hispanic youth less: hispanic versus non-hispanic youth
	Ethnicity	–	F	CGAS	Clinician	Post	▲	BBT	▼	ARC	B	Greater: hispanic versus non-hispanic less: hispanic versus non-hispanic
Brent (2020)	Ethnicity	–	S	PARS	Clinician	8 m FU		BBT	▼	ARC	A	Less: hispanic versus non-hispanic
	Comorbid (Internal)	CDRS-R	R	PARS	Clinician	8 m FU	▲	BBT		ARC	A	Greater: youth without depression at baseline no difference: youth with depression at baseline
Schwartz (2023)	Ethnicity	–	S	KSADS-PL	Clinician	4 m / 8 m FU	▲	BBT	▼	ARC	B	Greater: hispanic youth
	Ethnicity	–	R	SCARED-P/C	Parent & Child	4 m / 8 m FU	▲	BBT	▼	ARC	B	Greater: hispanic youth
	Ethnicity	–	F	SDQ-C /CBQ-P	Parent & Child	4 m / 8 m FU	▲	BBT	▼	ARC	B	Greater: hispanic youth
	Depressive symptoms	MFQ-C	F	SDQ-C	Child	4 m / 8 m FU	▲	BBT		ARC	A	Greater: with depressive symptoms no difference: without depressive symptoms
	Depressive symptoms	MFQ-C	F	CBQ-P	Parent	4 m / 8 m FU	▲	BBT		ARC	A	Greater: without depressive symptoms no difference: with depressive symptoms

Pattern refers to ***A: Simple interaction***: Moderator shows DTE in one condition or subgroup; ***B: Crossover interaction:*** Moderator shows an opposite effect in *different* condition; ***C: Opposite Crossover interaction:**** Moderator shows an opposite effect in the same condition; and ****D: Conditional interaction:*** Moderator has the same direction of effect in *different* conditions.

**Table 3:** Effect direction plot of moderator variables significantly associated with treatment outcome in active non-CBT control conditions

Active non-CBT controls													
CBT versus TAU													
Study author	Examined moderator	Measure	Index	Measure	Source	Timing of assessment	DTE	CBT		DTE	Control	Pattern	Interpretation of improvement: greater improvement, no difference, or less improvement
Ginsburg (2020)	Age	–	S	ADIS-C/P	Comb	Post	▲	M-CBT			TAU	A	Greater: older youth (ave 14.1 years, 1*SD* above *M* = 10.9)
	Primary disorder	ADIS-C/P	S	ADIS-C/P	Comb	Post	▲	M-CBT			TAU	D	Greater: with a social anxiety diagnosis
	Primary disorder		S	ADIS-C/P	Comb	Post		M-CBT		▲	TAU		Greater: without a social anxiety diagnosis
	Baseline anxiety	SCARED	F	CGI-S	Clinician	Post	▲	M-CBT			TAU	D	Greater: with higher baseline anxiety
	Baseline anxiety		F	CGI-S	Clinician	Post		BBT		▲	TAU		Greater: with lower baseline anxiety
Nordh (2022)	Internalising symptoms	RCADS	R	LSAS-CA	Child	3 m FU	▲	I-CBT			I-SUPP	A	Greater: higher internalising scores no difference: lower internalising scores
Smarason (2023)	Baseline anxiety	PARS	R	CGI-S	Clinician	Post		C-CBT		▼	TAU	A	Less: higher baseline anxiety
Kim (2020a)	Age	–	R	PARS	Clinician	Post	▲	BIACA			TAU	A	Greater: age group (10–13 years) no difference: age groups (6–9 and 13–16)
	Age	–	F	CGI	Clinician	Post	▲	BIACA			TAU	A	Greater: age group (10–13 years) no difference: age groups (6–9 and 13–16)
**CBT versus Alternative Treatment**													
Wallace (2017)	Number of AD	PARS	R	PARS	Clinician	Post	▲		ICBT		CCT	A	Greater: more than one anxiety disorder
	Number of AD	PARS	R	PARS	Clinician	Post			ICBT	▲	CCT	A	Greater: one anxiety disorder
	Parent education	Self report	R	PARS	Clinician	Post	▲		ICBT		CCT	A	Greater: higher likelihood of college education
	EMA sleep variables	Sleep diary	R	PARS	Clinician	Post	▲		ICBT		CCT	A	Greater: more sleep-related problems
	Negative events (IP)	Brief interview	R	PARS	Clinician	Post	▲		ICBT		CCT	A	Greater: more interpersonal negative events

												t
Lebowitz (2020)	Baseline anxiety	SCARED-C	R	SCARED-C	Child	Post	▲	ICBT		SPACE	A	Greater: higher baseline severity no difference: lower baseline severity
	Parental stress/negativity	Coded	R	SCARED-P	Parent	Post		ICBT	▲	SPACE	D	Greater: higher parental negativity
	Parental stress/negativity	Coded	R	SCARED-P	Parent	Post	▲	ICBT		SPACE		Greater: lower parental negativity
	Child Oxytocin (OT)	Medical	R	SCARED-P	Parent	Post		ICBT	▲	SPACE	A	Greater: higher levels of salivary oxytocin no difference: lower levels of salivary oxytocin
Zilcha-Mano (2021)	Family accom-modation	FASA	R	SCARED-P	Parent	Post		ICBT	▲	SPACE	A	Greater: Agreement on High/Low accommodation No difference: Agree on Moderate accommodation
	Child oxytocin (OT)	Medical	R	SCARED-C	Child	Post	▲	ICBT		SPACE	D	Greater: higher baseline OT + decrease in OT (child is agent of change)
	Child oxytocin (OT)	Medical	R	SCARED-P	Parent	Post		ICBT	▲	SPACE		Greater: higher baseline OT + increase in OT (mother is agent of change)

Pattern refers to ***A: Simple interaction***: Moderator shows DTE in *one* condition or subgroup; ***B: Crossover interaction:*** Moderator shows an opposite effect in *different* conditions; ***C: Opposite Crossover interaction:**** Moderator shows an opposite effect in the same condition; and ****D: Conditional interaction:*** Moderator has the same direction of effect in *different* conditions.

**Table 4:** Effect direction plot of moderator variables significantly associated with treatment outcome in active CBT control conditions

Active CBT controls												
CBT versus CBT												
Study author	Examined moderator	Measure	Index	Measure	Source	Timing of assessment	DTE	CBT	DTE	Control	Pattern	Interpretation of improvement: greater improvement, no difference, or less improvement
Bodden (2008)	Parental anxiety	ADIS-A	R	SCARED	Child	Pre to 3 m FU	▲	ICBT		FCBT	D	Greater: parent with an anxiety disorder
	Parental anxiety	ADIS-A	R	SCARED	Child	Pre to 3 m FU		ICBT	▲	FCBT		Greater: parent without an anxiety disorder
Maric (2018)	ADHD Symptoms	CBCL	R	ADIS-C/P	Comb	1 yr FU		ICBT	▲	FCBT	A	Greater: higher adhd symptoms no difference: lower ADHD symptoms
Kendall (2008)	Parent anxiety	ADIS-IV-L	R	MASC	Child	Pre/post to 1 yr FU	▼	ICBT		FCBT FESA	A	Less: child with an anxious father no difference: child with an anxious mother
Suveg (2009)	Sex	–	F	CBCL-P	Parent	Post to 1 yr FU	▲	ICBT		FCBT FESA	A	Greater: female social skills no difference: male social competence
	Sex	–	F	CBCL-P	Parent	Post to 1 yr FU		ICBT	▲	FCBT FESA	A	Greater FCBT: male school performance no difference: female school performance
Puleo (2011)	Autism symptoms	SRS-P	S	ADIS-C/P	Comb	Post	▼	ICBT		FCBT	A	Less: moderate autism symptoms no difference: no autism symptoms
Vaclavik (2017)	Parental acculturation	SMAS-DSI	R	RCMAS	Parent	Post		GCBT	▲	FCBT	D	Greater: High level of acculturation
	Parental acculturation	SMAS-DSI	R	RCMAS	Parent	Post	▲	GCBT		FCBT		Greater: low level of acculturation
Villabo (2018)	Primary diagnosis	ADIS-C/P	S	ADIS-C/P	Comb	Post		ICBT	▲	GCBT	A	Greater: with a social anxiety disorder (SoAD) No difference: with GAD or SAD
Helland (2022)	Emotion regulation	BRIEF	R	MASC	Parent	Post	▲	ICBT		GCBT	D	Greater: low emotional control
	Emotion regulation	BRIEF	R	MASC	Parent	Post		ICBT	▲	GCBT		Greater: high emotional control
Wergeland (2014)	Primary diagnosis	ADIS-C/P	R	SCAS-P	Parent	Post/1 yr FU	▲	ICBT		GCBT WL	A	Greater: with generalised anxiety disorder (GAD)
	Primary diagnosis	ADIS-C/P	R	SMFQ	Parent	Post	▲	ICBT		GCBT WL	A	Greater: with generalised anxiety disorder (GAD)
	Primary diagnosis	ADIS-C/P	R	SMFQ	Parent	1 yr FU		ICBT	▲	GCBT	A	Greater: with social anxiety disorder (SoAD)
Bjaastad (2023)	Treatment adherence	TASC-T	S	ADIS-C/P	Comb	LT FU (M = 3.9yrs)		ICBT	▲	GCBT	A	Greater: higher levels of adherence
	Therapeutic alliance	TASC-C/T	S	ADIS-C/P	Comb	LT FU (M = 3.9yrs)		ICBT	▲	GCBT	A	Greater: higher therapist-rated alliance with youth

												.	
Wood (2009)	Age	-	R	Composite	Comb	1 yr FU		ICBT	▲	FCBT	A	Greater: age group (10–13 years) No difference: age group (6–9 years)	
Wright and Woods (2020)	Phobia type	ADIS-C/P	S	BAT	Child	6 m FU	▲	OST		ICBT	A	Greater: with vomit phobia no difference: animal, BII, other	
**CBT versus Augmented or Modified CBT**													
Barrett (1996)	Age	–	S	ADIS-C/P	Comb	Post/12 m FU		ICBT	▲	CBT+FAM WL	A		Greater: age group (7–10) no difference: age group (11–14)
	Sex	–	S	ADIS-C/P	Comb	Post/12 m FU		ICBT	▲	CBT+FAM WL	A		Greater: female youth no difference: male youth
Silverman et al. (2024)	Parental reinforcement	PRBI	R	RCMAS-P	Parent	Post		ICBT	▲	CBT+REINF	A		Greater: higher baseline negativity scores
	Parental acceptance	PRBI	R	RCMAS-P	Parent	Post		ICBT	▲	CBT+REL	A		Greater: higher baseline acceptance scores
Patriarca (2022)	Parental acculturation	DSI	R	RCMAS-P/C	Parent+Child	Post/12 m FU		ICBT	▲	CBT+REINF	D		Greater: lower levels of parent acculturation
	Parental acculturation	DSI	R	RCMAS-P/C	Parent+Child	12 m FU	▲	CBT+REL		CBT+REINF			Greater: higher levels of parent acculturation
Liber (2008)	Primary diagnosis	ADIS-IV-C/P	F	CBCL-P	Parent	Post		ICBT+PPE	▲	GCBT+PPE	D		Greater: with a social anxiety disorder (SoAD)
	Primary diagnosis	ADIS-IV-C/P	F	CBCL-P	Parent	Post	▲	ICBT+PPE		GCBT+PPE			Greater: without a social anxiety disorder (SoAD)
Liber et al. (2008)	Therapeutic alliance	TPOCS-A	S	ADIS-C/P	Comb	Post	▲	ICBT+PPE		GCBT + PPE	A		Greater: higher child alliance scores
Manassis (2002)	Comorbid (Anxiety)	SASC	R	MASC-C	Child	Post	▲	ICBT+PPE		GCBT+PPE	A		Greater: High vs low social anxiety symptoms
Hardway (2015)	Age	–	R	CDI	Child	Post/3 m FU	▲	ICBT		ICBT+FAM WL	A		Greater: age group (11–14) No difference: older age group (15-18yrs)
Elkins (2016)	Baseline fear	ADIS-C/P	R	PDSS-C	Child	Post	▲	ICBT+FAM		WL	A		Greater: moderate fear scores (-1*SD*) No difference: high fear scores (+ 1*SD*)
	Baseline avoidance	ADIS-C/P	R	PDSS-C	Child	Post	▲	ICBT+FAM		WL	A		Greater: moderate avoidance scores (-1*SD*) no difference: high avoidance scores (+ 1*SD*)
Storch (2022)	Age	–	R	PARS	Parent	Post		ICBT	▲	BIACA TAU	A		Greater BIACA: age group (12–13) no difference BIACA: younger youth
	Comorbid (internalising)	CBCL	R	PARS	Parent	Post	▼	ICBT	▲	BIACA TAU	B		Greater BIACA: lower baseline internalising scores less ICBT: higher baseline internalising scores
	Comorbid (externalising)	CBCL	R	PARS	Parent	Post	▼	ICBT	▲	BIACA TAU	B		Greater BIACA: lower baseline ADHD symptoms less ICBT: higher baseline ADHD symptoms

Study author	Examined moderator	Measure	Index	Measure	Source	Timing of assessment	DTE	CBT	DTE	Control	Pat tern	Interpretation of improvement: greater improvement, no difference, or less Improvement
	Comorbid (autism)	SRS-P	R	PARS	Parent	Post	▼	ICBT		BIACA TAU	D	Less: moderate to high versus low RRB scores
	Comorbid (autism)	SRS-P	R	PARS	Parent	Post		ICBT	▼	BIACA TAU		Less: high RRB scores
Hollocks (2023)	Decision-making	IGT	R	PARS	Parent	Post		ICBT	▲	BIACA TAU	A	Greater BIACA: higher/better decision-making
deJong (2023)	Parental anxiety	FQ	R	ADIS-C/P	Comb	Post		TGE	▼	PGE MGE	A	Less PGE: high baseline parental anxiety
Farrell (2018)	Age	-	R	ADIS-C/P	Comb	Post	▼	OST+DCS		OST+PBO	A	Less: age group (11–14 years) no difference: age group (7–10 years)
	Age	-	R	CGI-S	Clinician	1 m FU	▲	OST+DCS		OST+PBO	A	Greater: age group (7–10 years) No difference: age group (11–14 years)
	Age	-	F	CGAS	Clinician	3 m FU	▼	OST+DCS		OST+PBO	A	Less: age group (11–14 years) no difference: age group (7–10 years)
Caron (2020)	Therapist efficacy	Self-rated	R	CGI-S	Nurse	Post	▲	CALM		CALM-R	A	Greater: higher nurse self-efficacy
	Therapist efficacy	Self-rated	F	CGAS	Nurse	3 m FU	▲	CALM		CALM-R	A	Greater: higher nurse self-efficacy
**CBT versus combination**												
Compton (2014)	Primary diagnosis	ADIS-C/P	R	PARS	Clinician	Post	▲	ICBT COMB	▲	SRT PBO	D	Greater SRT and COMB: with a social anxiety diagnosis (SoAD)
	Primary diagnosis	ADIS-C/P	R	PARS	Clinician	Post	▲	ICBT COMB		SRT PBO	A	Greater COMB: with a generalised anxiety diagnosis (GAD)
	Primary diagnosis	ADIS-C/P	R	PARS	Clinician	Post	▲	ICBT COMB		SRT PBO	A	Greater COMB: with a separation anxiety diagnosis (SAD)
Gonzalez (2015)	Parental anxiety	STAI	R	PARS	Clinician	Post		ICBT COMB	▲	SRT PBO	A	Greater SRT: high parental anxiety
Halldorsdottir (2015)	Comorbid (externalising)	ADIS-C/P	S	ADIS-C/P	Comb	Post	▼	ICBT COMB		SRT PBO	A	Less: Comorbid ADHD diagnosis no difference: Youth with anxiety disorders only
Sanchez (2019)	Sex	-	F	CAIS	Parent	Post	▲	ICBT COMB	▲	SRT PBO	D	Greater SRT and COMB: male youth no difference: female youth
Taylor (2018)	Ethnicity	-	R	SCARED-P	Parent	Post	▼	ICBT COMB		SRT PBO	A	Less ICBT: hispanic versus non-hispanic ethnicity
	Ethnicity	-	R	PARS	Clinician	Post		ICBT COMB	▼	SRT PBO	A	Less SRT: hispanic versus non-hispanic ethnicity

Study author	Examined moderator	Measure	Index	Measure	Source	Timing of assessment	DTE	CBT	DTE	Control	Pattern	Interpretation of improvement: greater improvement, no difference, or less improvement
Taylor (2018)	Parental psy-chopathology	BSI	R	SCARED-C	Child	Post		ICBT COMB	▲	SRT PBO	A	Greater SRT: high global psychopathology scores
	Parental psy-chopathology	BSI	R	SCARED-P	Parent	Post		ICBT COMB	▲	SRT PBO	A	Greater SRT: high global psychopathology scores
	Parental psy-chopathology	BSI	R	PARS	Clinician	Post	▼▲	ICBT COMB	▲	SRT PBO	C	Greater SRT and COMB: high global psychopathology scores less ICBT: high global psychopathology scores
	Primary diagnosis	ADIS-C/P	R	SCARED-P	Parent	Post	▼	ICBT COMB		SRT PBO	A	Less ICBT: with a selective mustism diagnosis (SM)
	Primary diagnosis	ADIS-C/P	R	PARS	Clinician	Post	▼	ICBT COMB		SRT PBO	A	Less COMB: with a selective mustism diagnosis (SM)
	Comorbid (anxiety)	SCARED-C	R	SCARED-P	Parent	Post	▲	ICBT COMB	▼	SRT PBO	B	Greater COMB: higher separation anxiety symptoms less SRT: higher separation anxiety symptoms
	Comorbid (anxiety)	SCARED-C	R	PARS	Clinician	Post	▲	ICBT COMB		SRT PBO	A	Greater COMB: Higher separation anxiety symptoms
	Comorbid (anxiety)	SCARED-P	R	SCARED-C	Child	Post	▲	ICBT COMB		SRT PBO	A	Greater COMB: higher panic scores
	Comorbid (anxiety)	SCARED-P	R	SCARED-P	Parent	Post	▼	ICBT COMB		SRT PBO	A	Less ICBT: higher panic scores
	Comorbid (anxiety)	SCARED-P	R	SCARED-P	Parent	Post	▲	ICBT COMB	▼	SRT PBO	C	Greater ICBT: higher school phobia less SRT: higher school phobia
	Comorbid (internalising)	MFQ-C	R	SCARED-C	Child	Post	▲▲	ICBT COMB		SRT PBO	D	Greater ICBT and COMB: higher depressive scores
	Comorbid (externalising)	CBCL	R	SCARED-C	Child	Post		ICBT COMB	▲	SRT PBO	A	Greater SRT: higher thought problem scores
	Comorbid (externalising)	CBCL	R	SCARED-P	Parent	Post	▼	ICBT COMB		SRT PBO	A	Less ICBT: higher rule-breaking scores
	Coping ability	CQ	R	SCARED-C	Child	Post	▲	ICBT COMB		SRT PBO	A	Greater ICBT: Higher/better coping scores

Pattern refers to ***A: Simple interaction***: Moderator shows DTE in *one* condition or subgroup; ***B: Crossover interaction:*** Moderator shows an opposite effect in *different* conditions; ***C: Opposite Crossover interaction:**** Moderator shows an opposite effect in the same condition; and ****D: Conditional interaction:*** Moderator has the same direction of effect in *different* conditions.

##### Time Invariant Moderators

1. *Age* was the most frequently investigated moderator, with significant associations with DTEs reported in seven studies. In the *active non-CBT control* category, Ginsburg et al. (2020) found that older children (average age 14) benefited more from modular CBT compared to treatment as usual (TAU). Kim et al., (2020a, 2020b) reported that children aged 10–13 years showed greater improvements in anxiety and functioning from BIACA compared to both younger (6–9 years) and older (13–16 years) age groups. Despite sharing the same control condition, these findings diverged in terms of which age group benefited most.

In the *active CBT control* category, Storch et al. (2022) found that children aged 12–13 years benefited more from BIACA than younger children in their sample (7–11 years), when compared to standard child CBT. Wood et al. (2009) showed that family CBT was more effective for children aged 10–13 years than for younger children in their sample (6–9 years). Despite examining different CBT variants, the replication across two independent trials, shared comparator, and consistent direction of effect suggest a preliminary trend that older pre-adolescents may benefit more from certain CBT variants compared to individual CBT than younger age groups. This trend is challenged by Hardway et al. (2015)**,** who found that children aged 11–14 years experienced greater treatment response following modified standard individual CBT for child phobias compared to when the same treatment was augmented with a family module, than adolescents (aged 15–18 years). In contrast to these findings Barrett et al. (1996) showed that augmenting standard CBT with a family module benefited younger children (7–10 years), but not older children (11–14 years), compared to CBT alone. Farrell et al. (2018) reported that older youths (11–14 years) had worse outcomes when CBT was augmented with D-Cycloserine (DCS) compared to placebo, while younger children (7–10 years) showed better outcomes with DCS at 1-month follow-up. These findings indicate inconsistency in the direction of age-related moderation effects across these two studies and intervention types.

2. *Sex* emerged as a significant moderator in 3 of the 20 investigations, all within the *active CBT control* category: Suveg et al. (2009) found that girls demonstrated greater social competence in the individual CBT condition compared to family CBT or TAU (FESA), with no differences observed for boys on the same measure. Conversely, boys showed improved school performance in the family CBT condition, while no differences were found for girls. In contrast, Barrett et al. (1996) reported that augmenting standard CBT with a family module benefited girls more than CBT alone, with no significant differences between conditions for boys.

In the CAMS trial (Walkup et al., 2008), Sanchez et al. (2019) found that boys showed greater functional improvement in the medication (sertraline; SRT) and combined CBT+SRT conditions compared to CBT alone. Girls, however, showed no significant differences across conditions. Despite sharing the same control condition, these findings were not consistent in effect direction, intervention types, or outcome measures, and no pattern was replicated across studies. Therefore, sex does not meet the criteria for consistent and robust moderation.

3. *Hispanic ethnicity* was associated with differential treatment effects in four studies across two trials. In *inactive control* comparisons, Hispanic youth receiving Brief Behavioural Therapy (BBT) showed significantly greater treatment response than those receiving Assisted Referral to Care (ARC), and also outperformed non-Hispanic youth in both groups on anxiety and functional outcomes (Brent et al., 2020; RobinWeersing et al., 2017). Despite sharing the same control condition, findings were not consistent in effect direction across the different outcome measures, and all three studies were derived from the same trial dataset.

In the *active CBT control* category, secondary analyses of the CAMS trial (Walkup et al., 2008) showed that Hispanic youth showed less improvement than non-Hispanic youth following child CBT, based on independent evaluator (IE) ratings. In contrast, parent reports indicated that Hispanic youth experienced less improvement following sertraline compared to CBT, combined treatment, and placebo, highlighting variability in perceived outcomes across informants (Taylor et al., 2018). Together, although these findings suggest that Hispanic ethnicity may influence treatment response, the direction and nature of effects varied across studies and informants. Therefore, Hispanic ethnicity does not meet the criteria for consistent and robust moderation.

##### Time-Variant Moderators

4. *Primary anxiety diagnosis* (disorder type) was the most frequently investigated clinical moderator, with significant associations observed in six studies. In the *active non-CBT control* category, Ginsburg et al. (2020) found that children with Social Anxiety Disorder (SoAD) were more likely to remit their anxiety diagnosis following modular CBT, compared to children without SoAD who showed greater remission following supportive therapy.

In comparisons involving *active CBT controls*, Villabo et al. (2018) found that Group CBT was more effective than individual CBT for youth with SoAD, showing greater remission at post-treatment, with no significant differences for youth with Generalised Anxiety Disorder (GAD) or Separation Anxiety Disorder (SAD). Similarly, Group CBT led to better functional outcomes at post-treatment (Liber et al., 2008) and improved treatment response at 1-year follow-up for youth with SoAD (Wergeland et al., 2014). Youth without SoAD responded better to individual CBT on functional outcomes at post-treatment (Liber et al., 2008), and youth with GAD showed greater treatment response with individual CBT post-treatment and 1-year follow-up (Wergeland et al., 2014). While these findings suggest a promising trend for the potential advantage of Group CBT, they were not consistently replicated across outcome measures and assessment points. Further support for diagnosis-specific effects comes from secondary analyses of the CAMS trial (Walkup et al., 2008), where treatments involving medication, either combined CBT+SRT or SRT alone, were more effective for youth with SoAD (Compton et al., 2014). For youth with GAD, SAD, and Selective Mutism (SM), only the combined treatment showed superior outcomes (Taylor et al., 2018). Although these two studies support the notion that medication-based treatments for youth with SoAD may be more beneficial, they were derived from the same trial dataset. Therefore, despite the observed trend, primary anxiety diagnosis does not meet the criteria for consistent and robust moderation.

5. *Baseline anxiety severity* was a significant moderator in four studies within the *active non-CBT control* category. Ginsburg et al. (2020) found that youth with higher anxiety severity showed greater functional improvement following modular CBT compared to TAU, while those with lower baseline severity responded better to supportive therapy. Similarly, Smarason et al. (2023) reported that youth with higher anxiety severity had poorer treatment response in the TAU condition compared to those receiving computer-assisted CBT. Lebowitz et al. (2020) found that individual CBT was more effective than SPACE (Supportive Parenting for Anxious Childhood Emotions) for youth with higher anxiety severity at post-treatment, while no significant differences were observed for those with lower anxiety baseline severity. All three studies showed a consistent direction of effect, favouring CBT-based interventions for youth with higher severity, despite effects based on different outcome measures. Therefore, baseline anxiety severity shows preliminary evidence of consistent moderation within a shared comparator control category, but does not meet the criteria of consistent and robust moderation.

6. *Depressive symptoms* were associated with differential treatment effects in four studies. In an *inactive control* comparison, Brent et al., (2020) found that youth without depressive symptoms at baseline benefited more from BBT than TAU at 8-month follow-up, based on anxiety outcomes. No significant differences between conditions were evident for youth with depressive symptoms. Based on the same trial, Schwartz et al. (2023) reported similar findings for youth without depressive symptoms on parent-reported functional outcomes. However, among youth with depressive symptoms, BBT was also more effective than TAU based on child-reported functional outcomes. Despite sharing the same control condition and direction of moderation effects, findings were not consistent across outcome measures and were derived from the same trial dataset. In the *active non-CBT control* category, Nordh et al. (2022) found that children with higher depressive symptoms showed greater response to therapist-guided internet-delivered CBT compared to an I-support TAU condition, with no significant differences for children with low depressive symptoms.

In the *active CBT control* category, secondary analyses from the CAMS trial (Walkup et al., 2008) indicated that youth with higher depressive symptoms benefited more from treatments including CBT, both CBT alone and combined CBT+SRT, based on child-reported anxiety measures at post-treatment (Taylor et al., 2018). Direct comparability is limited for these effects as they span different control categories; therefore, depressive symptoms do not meet the criteria for consistent and robust moderation.

7. *ADHD symptoms* were identified as significant moderators in two studies, both within the *active CBT control* category. Maric et al. (2018) found that for youth with higher ADHD symptoms, Family CBT was more effective than Individual CBT for anxiety outcomes at 1-year follow-up, with no differences observed for youth with lower symptom scores. Storch et al. (2021) reported that a modified CBT intervention (BIACA) was more effective for youths with lower ADHD symptoms at baseline, based on parent-reported anxiety outcomes. In contrast to Maric’s findings of little effect of individual CBT for children with higher ADHD symptoms, Storch et al. (2021) found that individual CBT negatively affected anxiety outcomes for children with higher baseline ADHD symptoms assigned to that condition. These two studies show some trend in individual CBT being less effective, even detrimental, for youth with higher comorbid ADHD symptoms, while alternative CBT formats (Family CBT or BIACA) may offer better outcomes. However, the contrasting direction of effect depends on the intervention format and symptom severity, and as such, ADHD symptoms do not meet the criteria for consistent and robust moderation.

8. *Autism* symptoms were associated with differential treatment effects in two studies, both within the *active CBT control* category. Puleo et al. (2011) found that children with moderate autism symptoms at baseline had a lower likelihood of anxiety remission when assigned to individual CBT compared to family CBT. No group differences were observed for children without autism symptoms. Storch et al. (2021) found that children with moderate to high autism symptoms experienced worse anxiety outcomes post-treatment when assigned to individual CBT compared to the modified BIACA condition. However, children with high autism scores also showed poorer anxiety outcomes in the BIACA condition. These two studies show some trend in individual CBT being less effective for youth with moderate to high comorbid Autism symptoms. However, given the variation in outcomes and effects in other CBT formats, Autism symptoms do not meet the criteria for consistent and robust moderation.

9. *Parental psychopathology* at baseline was significantly associated with differential treatment outcomes in 5 of 10 investigations, all within the *active CBT control* category. When child CBT was compared to family CBT, Bodden et al. (2008) found that when a parent had an anxiety disorder, children benefited more from individual CBT at post-treatment and 3-month follow-up, whereas family CBT was relatively more effective for children whose parents did not have an anxiety disorder. Kendall et al. (2008) observed a different pattern at 1-year follow-up: children with an anxious father (but not mother) showed significantly less improvement in anxiety symptoms following individual CBT compared to those receiving family CBT or FESA. In a trial of modified exposure treatment, de Jong et al. (2023) found that youth with highly anxious parents had worse outcomes when exposed to parent-guided sessions, compared to therapist-guided or minimally guided sessions. Examining the same outcomes across different occasions, these findings suggest a tentative trend: when parental anxiety is high, reduced parental involvement may sometimes be more effective; however, the direction of effects is not consistent across studies or timepoints. In the CAMS trial (Walkup et al., 2008), youth whose parents had high anxiety showed the greatest treatment response in the medication (sertraline; SRT) condition, compared to CBT, combined treatment, or placebo (Gonzalez et al., 2015). Similarly, Taylor et al. (2018) found that SRT was most effective, based on child, parent, and clinician reports, for youth whose parents had high levels of global psychopathology. These findings suggest that medication-based treatments may be more effective when parental psychopathology is high, but both results were derived from the same trial dataset. Taken together, parent psychopathology does not meet the criteria for consistent and robust moderation and cannot prescribe format selection.

10. *Parent acculturation* was significantly associated with differential treatment response in both studies that examined this moderator. Vaclavik et al. (2017) found that when parents reported high levels of acculturation, family CBT led to greater parent-reported treatment response at post-treatment compared to peer-involved Group CBT. In contrast, Group CBT was more beneficial when parent acculturation was low. Similarly, Patriarca et al. (2022) reported that CBT augmented with a parent relationship skills module targeted at reducing psychological control (CBT+REL) was more effective than CBT with a parent reinforcement skills module targeted at reducing negative reinforcement (CBT+REINF) when acculturation was high. Conversely, CBT+REINF was more effective at low levels of acculturation. These two studies show a consistent trend: when parent acculturation is high, treatment response is better following relational/family-oriented CBT formats. Given differences in optimal formats when acculturation is low, parental acculturation does not yet meet the criteria for consistent and robust moderation.

11. *Child oxytocin* was significantly associated with differential treatment outcomes in both studies within the *active non-CBT control* condition. Lebowitz et al. (2020) found that children with higher levels of salivary oxytocin benefited more from SPACE (Supportive Parenting for Anxious Childhood Emotions) than from individual CBT at post-treatment. Similarly, for children with higher levels of salivary oxytocin, Zilcha-Mano et al. (2023) found that SPACE was more effective than individual CBT when oxytocin levels increased during treatment and the mother was the agent of change. Conversely, when oxytocin levels decreased during treatment and the child was the agent of change, individual CBT was more effective. No significant differences between conditions were observed at lower levels of oxytocin. These two studies show a similar trend that higher levels of salivary oxytocin were associated with better outcomes in SPACE compared to individual CBT, particularly when parental involvement was central and oxytocin levels increased during treatment. However, because these findings are derived from a single trial, they do not meet the criteria for consistent and robust moderation.

## Discussion

This review systematically examined differential treatment effects (DTEs), that is, those interactions between the moderator variable(s) and treatment condition that indicate which treatment is better for a child with a certain characteristic (Kraemer, 2016). This is in contrast with the mainstream understanding of moderation, or variables that interact with other variables to affect treatment outcomes. As such, this systematic review updates the literature synthesis in the field of childhood anxiety research. Since there have been no previous syntheses in this domain that use these methodologically robust and explicit criteria, our approach was necessarily exploratory and narrative in nature.

### Potential Moderators of Differential Treatment Effects

This systematic review aimed to identify moderator variables that demonstrate sufficient consistency and robustness to inform clinical decision-making. Of the eleven moderators showing significant DTEs in multiple studies, three (*Hispanic ethnicity*, *depressive symptoms*, and *child oxytocin levels)* came from the same underlying trial dataset. As they do not represent independent replications across different samples, the DTEs cannot be considered consistent or robust.

Of the remaining eight moderators evaluated across different studies within the same control condition, three (*sex*, *ADHD symptoms*, and *Autism symptoms)* were too heterogeneous to support definitive conclusions. Five moderators (age, Social Anxiety disorder, baseline anxiety, parent psychopathology, and parent acculturation) showed preliminary trends in differential treatment effects (DTEs), though findings remain tentative and should not be interpreted as prescriptive. For example, DTEs for parent psychopathology were observed in 3 studies within the same control category, suggesting that when parental psychopathology is high, interventions with reduced parental involvement may sometimes yield better outcomes. However, these findings are inconsistent and must be interpreted with caution, given substantial variability in CBT protocols. Rather than indicating a global recommendation, these trends highlight areas for further investigation into how parental factors interact with specific CBT variants.

Findings for these five moderators are partially aligned with earlier reviews of moderators in the field (Norris & Kendall, 2020; Zhou et al., 2025). The narrative review reported mixed findings for moderators such as age, baseline anxiety severity, and parent psychopathology, with somewhat more consistent support for primary diagnosis (Social Anxiety Disorder) (Norris & Kendall, 2020), and noted that methodological variability across studies limited generalisability. The meta-analysis by Zhou et al. (2025) found some support for moderators of CBT effectiveness, primarily in studies of universal prevention. For treatment-focused CBT, there was limited support for age, facilitator contact time, and delivery formats as moderators, though the authors advised caution due to heterogeneity, limited data, and publication bias.

By organising studies within the same control category and applying strict inclusion and measurement criteria consistently, this review reduced confounding variability and improved interpretability of DTEs, offering greater precision and clinical relevance than broader-scope reviews with heterogeneous comparators and outcomes. In the absence of established criteria for consistent and robust DTE moderators, this review applied a set of logical, albeit conservative, standards to flag emerging trends, underscoring the need for the field to develop and adopt formal, empirically grounded benchmarks for evaluating moderator evidence as consistent and robust.

### Implications for Personalisation

Identifying subgroups with DTEs remains central to personalised and precision medicine. Subgroup analyses clarify how individuals with specific characteristics respond to a treatment versus an alternative (Alemayehu et al., 2018). Consistent with previous reviews, this systematic review found no reliable moderators of childhood anxiety outcomes (Herres et al., 2015; Nilsen et al., 2013). The lack of robust evidence has led some to question whether personalised treatment is possible at all. However, the absence of moderator evidence should not be mistaken for evidence of moderator absence (Wang & Ware, 2013). Moderators may exist and remain undetected due to some of the methodological limitations discussed in this review (for example, the narrow range of variables typically examined). Advancing personalisation may require expanding or combining attributes (Zilcha-Mano, 2019). Nevertheless, without moderators systematically associated with DTEs, a personalised approach to treatment selection remains out of reach (Norris & Kendall, 2020).

### Challenges and Future Research Directions

This review reflects on the challenges in identifying and evaluating DTEs and highlights key considerations for future research.

#### Design and Sample Size

Advancing empirical evidence requires a high level of specificity in the research questions being asked (Wang & Ware, 2013), which explicitly states hypotheses (primary or secondary) to clarify whether statistical interaction tests are pre-specified or post hoc (Van Hoorn et al., 2017). Another major challenge is that too few studies are designed a priori to test interaction effects, and most are underpowered to detect heterogeneity in treatment effects across subgroups (Brankovic et al., 2019). No single study in this review provided strong moderator evidence, indicating the need for larger and better-powered samples (Hancock & Kent, 2022). Recognising this need and not leveraging existing datasets represents a missed opportunity (Kraemer, 2024). Increasingly, researchers are advocating for pooled RCT data to examine treatment moderators through individual patient data (IPD) meta-analysis (Bertie et al., 2024a, 2024b) as a means to address power constraints. Future trials should be designed with data harmonisation in mind to facilitate data aggregation.

#### Moderator Variables

Defining the role of and expected influence of moderator variables a priori ensures alignment between the language, theoretical argument, and resulting choice of statistical analyses (Andersson et al., 2014). The inconsistent findings in this review may reflect an overreliance on single moderators to predict superior treatment outcomes (Zilcha-Mano, 2019), whereas combinations of factors may better capture individual differences and yield greater clinical utility (Kraemer, 2016). Also, six potential moderators were time-variant variables. Although assessed at baseline, DTEs (e.g., social anxiety disorder and parental psychopathology) may be influenced by intervening factors, including potential reverse effects (where marked improvement in the child’s mental health may alleviate anxiety symptoms in their parents). This highlights the importance of understanding the clinical motivation and the role of the moderator variable.

#### Statistical Analyses

Outcome choice (remission, response, or functioning) influences the choice of assessment and interpretation of interaction effects (Wang & Ware, 2013). Similarly, different statistical models (linear vs logistic regression) operate on different scales and estimate different effect measures (Brankovic et al., 2019). The common practice of testing one moderator at a time may stem from traditional training in psychological statistics, which often emphasises basic moderator concepts and conventional regression techniques over more advanced regression or exploratory methods (Aiken et al., 2009). In this review, only three studies employed alternative approaches such as a combined moderator (Wallace et al., 2017), machine learning (Lebowitz et al., 2020), and correspondence analysis (Kim et al., 2020a, 2020b). Advanced methods may support a shift from explanatory to predictive modelling, essential for personalisation (see Zilcha-Mano, 2019, for further discussion).

#### Reporting and Interpretation

Insufficient and unclear reporting hampers the interpretation and evaluation of definitive DTE findings. Most studies in this review had some risk of bias from either the quality of the original trial or the moderator analysis, often due to inadequate reporting and the absence of power analyses. Preregistration of trial protocols and clear specification of planned moderator analyses, plus adherence to established methodological guidelines, can reduce the risk of bias and improve the quality of studies.

The underreporting of null results persists, particularly of non-significant DTE tests (Schneider et al., 2015). Comprehensive reporting of significant and null findings reduces publication bias (Zilcha-Mano, 2019) and clarifies why DTEs appear absent or inconsistent. In this review, inconclusive results for 35 moderator variables lacked the methodological or statistical detail necessary for interpretation and do not contribute to the science supporting personalisation. For example, null or inconclusive DTEs may indicate a predictor variable (not a moderator), or reflect underpowered trials (Type II error). Importantly, statistical significance alone does not automatically contribute to the evidence base (Andersson et al., 2014). Transparent reporting is essential for advancing the field, allowing researchers to validate, replicate, and ultimately discard redundant moderator variables (Brankovic et al., 2019).

Collectively, these challenges amplify the call for a standardised approach to analysis, synthesis, and reporting of DTE research to enable cumulative knowledge across significant, null, and inconclusive results. There is considerable potential for a rigorous research agenda that leverages recent theoretical and technological advances to identify moderators of CBT outcomes (Bertie & Hudson, 2021). Theory development requires a consensus criterion for ‘definitive’ moderators, while statistical development requires big data and machine learning solutions (Wright & Woods, 2020) to address persistent methodological challenges. A unified approach will permit meaningful cross-study comparisons and accelerate progress towards personalised interventions.

### Limitations of the Present Review

Several methodological factors limit the generalisability of our findings and warrant careful consideration. Restricting this review to English, peer-reviewed articles ensures a high standard of quality but may introduce language and publication bias (Zilcha-Mano, 2019). Conclusions drawn from moderator analyses are constrained by study quality: only 38% of moderator analyses were confirmatory, many were exploratory or post hoc analyses, often underpowered or using unvalidated measures.

In adopting a novel methodological approach, control conditions were categorised to reduce variability and enhance the interpretability of moderator results. However, few studies examined moderator variables across comparable treatment or control conditions. Grouping all active CBT conditions may have obscured nuanced distinctions between specific components (i.e., exposure), limiting insights into their relative effectiveness. Importantly, this limitation presents a broader challenge for interpreting DTEs, which extends beyond component-level differences. CBT protocols can vary substantially in structure, intensity, and emphasis on certain techniques. As such, moderator findings in one CBT protocol (e.g., one that uses minimal exposure) may not generalise to another that prioritises exposure-based strategies. This protocol heterogeneity within CBT complicates DTE interpretation and underscores the need for future research to examine protocol-level variability more systematically.

For this review, CBT was defined as interventions explicitly incorporating cognitive and/or behavioural strategies (typically psychoeducation, cognitive restructuring, and exposure-based techniques). Future research should compartmentalise CBT components (e.g., exposure, parental involvement) to delineate which elements contribute to moderation effects and for whom.

Due to methodological heterogeneity (outcome measures, timing of assessments, and reporters), DTEs could not be examined using meta-analysis. Additional limitations arise when stratification and interaction modelling use post-baseline factors, which compromises the benefits of baseline randomisation (Wang & Ware, 2013) and increases confounding. For instance, a moderator associated with baseline severity may divide the group into mild versus severe subgroups, with the latter group showing greater potential for positive response, misattributing the DTE to only one variable when multiple factors may contribute to the observed outcomes (Kraemer, 2016). According to current understanding and practice, this review retained 11 studies that examined post-baseline factors as potential moderators of DTEs, despite recommendations against this approach (Kraemer et al., 2016).

## Conclusion

This preregistered systematic review adhered to best practice guidelines and rigorous methods to strengthen its validity. Data were systematically extracted, organised, and evaluated by control condition (Karlsson & Bergmark, 2015), clarifying which treatments may be most effective for which subgroups, a comparative approach not previously documented. This review highlighted inconsistencies in the current application and interpretation of statistical interaction analyses, as well as their resulting implications for clinical research and decision-making, and introduced new considerations for interpreting DTEs.

Although comparing individual moderator variables and their treatment effects remains challenging, the search for moderators is essential to guide clinicians toward personalised interventions for children and young people. To date, no definitive moderators have been identified; whether due to true absence of moderators or methodological limitations, remains unclear. Before concluding the former, a more rigorous research agenda is needed to address persistent challenges in heterogeneity research and support future synthesis, including a classification framework for DTEs. In the interim, baseline variables such as age, a diagnosis of social anxiety disorder, baseline anxiety severity, parental psychopathology, and parent acculturation offer promising avenues for hypothesis-testing research, ideally with newer statistical approaches applied to larger datasets. In sum, this review raises awareness of the methodological advancements required to support future personalisation research and ultimately improve outcomes for children and adolescents with anxiety disorders.

## Supplementary Information

Below is the link to the electronic supplementary material.

Supplementary file1 (DOCX 113 kb)

## Data Availability

No datasets were generated or analysed during the current study.
