# Supplementary material for: Moving Beyond Moderation to Identify Differential Treatment Effects of Cognitive Behavioural Therapy for Childhood Anxiety: A Systematic Review
Source: Clin Child Fam Psychol Rev. 2026 Mar 6;29(3):452–76. doi: 10.1007/s10567-026-00564-8 (PMC13562111; doi:10.1007/s10567-026-00564-8)
Supplement: Supplementary file 1 [file 10567_2026_564_MOESM1_ESM.docx]

**Supplementary Materials**

Moving Beyond Moderation to Identify Differential Treatment Effects of Cognitive Behavioural Therapy for Childhood Anxiety: A Systematic Review

***Clinical Child and Family Psychology Review***

Authors: Bertie, Mackinnon, McDermott, O’Gradey-Lee, Kikas, Tomlinson, & Hudson

**A. SEARCH STRATEGY**

**Search Strings for Ovid PsycINFO, Ovid Embase, and PubMed/MEDLINE** [11 July 2025]

| **Search Criteria** | **OVID** | **PubMed** | **Search Hits** |
| --- | --- | --- | --- |
| Children and Adolescents | #1 | #1 | (child* OR teen* OR adolesc* OR youth OR “young pe*” OR juvenil* OR school-age* OR pre-adolesc* OR preadolesc* OR minor* OR paediatric* OR pediatric* OR “under 18”).mp. |
| Exclude adults | - | #2 and #3 | (adult or “older adult” or “over 18”).mp. |
| Anxiety disorders | #2 | #4 | (“anxiety disorder*” OR anxi* OR “general* anxiety” OR “social anxiety” OR “separation anxiety” OR “social phobi*” OR “specific phobi*” OR “selective mutism” OR “panic disorder” OR internali#ing OR “school refusal”).mp. |
| Exclude conditions not anxiety |  | #5 and #6 | (OCD or "obsessive compulsive" or PTSD or "posttraumatic stress" or "post-traumatic stress") |
| CBT interventions | #3 | #7 | (“cognitive behavio* therap*” OR “cognitive therap*” OR “behavio* therap” OR “exposure therap*” OR CBT).mp |
| RCTs | #4 | #4 | (“randomi* control* trial” OR “control* trial” OR “clinical trial” OR RCT OR randomi#ed OR randomi#ation OR cluster).mp |
| Combined | #5 | #5 | 1 and 2 and 3 and 4 |
| Exclude reviews |  |  | (protocol or review or “systematic review” or meta-analysis or “meta analysis”).mp. |
| Moderators | #6 | #6 | (moderate* or moderator or “moderat* analysis” or “moderator variable” or interact* or “interaction test*” or “effect modification” or subgroup* or “differential treatment effect”).mp. |
| Including moderators | #8 | #8 | 6 and 7 |

*Note:* Search fields: mp (multi-purpose) is a field tag used to search across multiple fields and typically includes for OVID databases: ab (abstract), ti (title), sh (subject headings), and kf (keyword heading words), id (key concepts), md (methodology), mf (mesh word). For other databases: title (ti), abstract (ab), subject headings (sh), keyword heading words (kf)

| Covidence  (Import) | PsycInfo | EMBASE | MEDLINE | TOTAL |
| --- | --- | --- | --- | --- |
| 1 | 678 | 1144 | 563 | 2385 |
| 2 | 145 | 276 | 122 | 543 |
| Total Imported | 823 | 1420 | 685 | **2928** |
|  |  |  |  |  |

**B. STUDY SELECTION**

**Summary of Study Selection and Coding**

| **IRRELEVANT STUDIES** | | | **UNAMBIGUOUS** | **AMBIGUOUS** | **Total** |
| --- | --- | --- | --- | --- | --- |
|  |  |  |  |  |  |
| P | 1 | Wrong sample | 73 | 16 | 89 |
|  | 2 | Medical/Physical | 152 | 0 | 152 |
|  | 3 | Not Anxiety Disorder | 439 | 4 | 443 |
| IC | 4 | Not a CBT intervention | 20 | 3 | 23 |
| O | 5 | Other outcomes | 69 | 8 | 77 |
| S | 6 | Not RCT | 46 | 22 | 68 |
|  | 7 | Prevention study | 53 | 4 | 57 |
|  | 8 | Protocol | 137 | 248 | 385 |
|  |  | - (clinical trials register) | 176 | -176 | 0 |
|  |  |  | **1165** | **129** | **1294** |

**C. CHARACTERISTICS OF TRIAL AND MODERATOR STUDIES INCLUDED IN THE SYSTEMATIC REVIEW AND ARRANGED BY CONTROL CONDITION**

| **Trial author** | **N** | **Age M** | **Age range** | **Intervention and Control conditions** | **Program Description** | **Control Category** | **Duration (sessions)** | **Moderator Study Author** | **Non-significant Moderators** | **Significant Moderators** |
| --- | --- | --- | --- | --- | --- | --- | --- | --- | --- | --- |
| Diego et al., 2024 | 86 | 10.1 | 8-12 | 1. Group CBT | SSL | Waitlist | 8 | Diego et al., 2024 | Sex |  |
|  |  |  |  | 2. Waitlist | - |  |  |  |  |  |
| Hayward et al., 2000 | 35 | 15.8 | - | 1. Group CBT | CBGT-A |  | 16 | Hayward et al., 2000 | Comorbid status (prior mood) |  |
|  |  |  |  | 2. Waitlist |  |  |  |  |  |  |
| Kendall et al., 1997 | 94 | - | 9-13 | 1. Child CBT | Coping CAT |  | 16 | Kendall et al., 1997 | Age |  |
|  |  |  |  | 2. Waitlist | - |  |  |  | Comorbid status |  |
| Silverman et al., 1999 | 56 | 10.0 | 6-16 | 1. Group CBT | Children & Parents |  | 8 | Silverman et al., 1999 | Age, Sex, Ethnicity |  |
|  |  |  |  | 2. Waitlist | - |  |  |  | Therapist pairing / Comorbid status |  |
| Beidel et al., 2000 | 67 | 10.5 | 8-12 | 1. Group CBT | SET-C | Inactive TAU | 12 | Beidel et al., 2000 | Age, Sex, Ethnicity |  |
|  |  |  |  | 2. TAU | Testbusters |  |  |  | Comorbid status |  |
| Ollendick et al., 2009 | 196 | 11.0 | 7-16 | 1. Individual CBT | OST |  | 1 | Ollendick et al., 2009 | Age, Sex, Country |  |
|  |  |  |  | 2. TAU | Educational Support (ES) |  |  |  | Primary diagnosis (SP) |  |
|  |  |  |  | 3. Waitlist |  |  |  | Halldorsdottir et al., 2016 | ADHD symptoms |  |
| Ollendick et al., 2019 | 58 | 14.3 | 12-16 | 1. Child CBT | ABMT |  | 10 | Ollendick et al., 2019 | Threat bias | Baseline anxiety |
|  |  |  |  | 2. TAU | ACT |  |  |  |  | Attention control |
| Weersing et al., 2017 | 185 | 11.3 | 8-17 | 1. Child CBT | BBT |  | 8-12 | Weersing et al., 2017 | Depressive symptoms | Ethnicity |
|  |  |  |  |  |  |  |  |  | Site |  |
|  |  |  |  | 2. TAU | ARC |  |  | Brent et al., 2020 |  | Ethnicity, Depressive symptoms |
|  |  |  |  |  |  |  |  | Schwartz et al., 2023 |  | Ethnicity, Depressive symptoms |
| Barrington et al., 2005 | 54 | 10.0 | 7-14 | 1. Child CBT | Manual/ Standard | Active non-CBT TAU | 12 | Barrington et al., 2005 | Parent anxiety (maternal) |  |
|  |  |  |  | 2. TAU | Variety* |  |  |  |  |  |
| Ginsburg et al., 2020 | 216 | 10.9 | 6-18 | 1. Child CBT | M-CBT |  | 12 | Ginsburg et al., 2020 | Sex, Ethnicity | Age |
|  |  |  |  | 2. TAU | Supportive Therapy (ST) |  |  |  | Primary diagnosis (GAD, SAD) | Baseline anxiety |
| Nordh et al., 2021 | 103 | 14.1 | 10-17 | 1. Child CBT | I-CBT |  | 10 | Nordh et al., 2022 | Age, Sex, Baseline anxiety | Depressive symptoms |
|  |  |  |  | 2. TAU | ISUPPORT |  |  |  | Previous treatment, Age of onset, |  |
|  |  |  |  |  |  |  |  |  | Maintaining factors (anticipatory anxiety, post-event processing, internal & external focus of attention, safety behaviours, family accommodation) |  |
| Storch et al., 2015b | 100 | 9.8 | - | 1. Child CBT | Camp-Cope-A-Lot | Active non-CBT TAU | 12 | Smarason et al., 2023 | Age, Sex, | Baseline anxiety |
|  |  |  |  | 2. TAU | Active standard care |  |  |  | Depressive symptoms, Externalising problems, Internalising problems |  |
|  |  |  |  |  |  |  |  |  | Caregiver strain, Child functioning |  |
|  |  |  |  |  |  |  |  |  | Primary Diagnosis (SoAD) |  |
| Storch et al., 2013 | 45 | 8.9 | 7-11 | 1. Child CBT | BIACA |  |  16 | Kim et al., 2019 |  | Age |
|  |  |  |  | 2. TAU | Active standard care |  |  |  |  |  |
| Storch et al., 2015a | 31 | 12.7 | 11-16 | 1. Child CBT | BIACA |  | 16 |  |  |  |
|  |  |  |  | 2. TAU | Active standard care |  |  |  |  |  |
| Wood et al., 2015 | 33 | 12.3 | 11-15 | 1. Child CBT | BIACA |  | 16 |  |  |  |
|  |  |  |  | 2. Waitlist |  |  |  |  |  |  |
| Silk et al., 2016 | 133 | 11.0 | 9-14 | 1. Child CBT | Coping CAT | Alternative non-CBT Treatment | 16 | Silk et al., 2016 | Age, Sex, Baseline anxiety |  |
|  |  |  |  | 2. Psychotherapy | CCT |  |  |  | Primary diagnosis, Comorbid status |  |
|  |  |  |  | Both at post-treatment | + Sleeping Tigers |  | 6-8 |  | Follow-up intervention status |  |
|  |  |  |  |  |  |  |  | Wallace et al., 2017 |  | Number of anxiety disorders |
|  |  |  |  |  |  |  |  |  |  | EMA sleep-related characteristics (3) |
|  |  |  |  |  |  |  |  |  |  | Interpersonal negative events |
|  |  |  |  |  |  |  |  |  |  | Parent education |
|  |  |  |  |  |  |  |  | Silk et al., 2019 | Age, Sex, Pubertal status |  |
|  |  |  |  |  |  |  |  |  | Treatment response |  |
| Lebowitz et al., 2019 | 124 | 10.5 | 7-14 | 1. Individual CBT | Manual/ Standard |  | 12 | Lebowitz et al., 2021 | Age, Sex, Primary diagnosis, Social skills | Parental Negativity |
|  |  |  |  | 2. Psychotherapy | SPACE (parent program) |  |  |  | Depressive symptoms / externalising symptoms | Child oxytocin |
|  |  |  |  |  |  |  |  |  | Parental anxiety, Parental depression | Baseline anxiety |
|  |  |  |  |  |  |  |  |  | Maternal acceptance and control |  |
|  |  |  |  |  |  |  |  |  | Family accommodation, Parental involvement |  |
|  |  |  |  |  |  |  |  |  | Parent oxytocin |  |
|  |  |  |  |  |  |  |  | Zilcha-Mano et al., 2020 |  | P/C Agreement on Accommodation |
|  |  |  |  |  |  |  |  | Zilcha-Mano et al., 2023 |  | Child oxytocin |
| Bodden et al., 2008 | 128 | 12.4 | 8-18 | 1. Child CBT | Manual/ Standard | Active CBT | 13 | Bodden et al., 2008 | Age | Parental anxiety |
|  |  |  |  | 2. Family CBT | Manual/ Standard |  |  | Maric et al., 2018 |  | ADHD symptoms |
| Kendall et al., 2008 | 161 | 10.3 | 7-14 | 1. Individual CBT | Coping CAT |  | 16 | Kendall et al., 2008 |  | Parental anxiety |
|  |  |  |  | 2. Family CBT | Coping CAT |  |  | Suveg et al., 2009 | Age | Sex |
|  |  |  |  | 3. TAU | FESA |  |  | Puleo et al., 2011 |  | Autism Symptoms |
| Pincus et al., 2015 | 57 | 15.4 | 12-17 | 1. Individual CBT | AIP |  | 6 | Hardway et al., 2015 |  | Age |
|  |  |  |  | 2. Individual CBT | AIP |  |  | Elkins et al., 2016 |  | Baseline fear and avoidance |
|  |  |  |  |  | + Parent Involvement (FAM) |  |  |  |  |  |
|  |  |  |  | 3. Waitlist | - |  |  |  |  |  |
| Silverman et al., 2019 | 240 | 9.8 | 7-16 | 1. Group CBT | Peer groups |  | 12-14 | Vaclavik et al., 2017 | Parental identification | Parental Acculturation |
|  |  |  |  | 2. Family CBT | Dyadic parent groups |  |  |  |  |  |
| Villabö et al., 2018 | 165 | 10.5 | 7-13 | 1. Individual CBT | Coping CAT |  | 14 | Villabö et al., 2018 | Primary diagnosis (SAD, GAD) | Primary diagnosis (SoAD) |
|  |  |  |  | 2. Group CBT | Coping CAT |  |  | Helland et al., 2022 |  | Emotion regulation |
|  |  |  |  | 3. Waitlist | - |  |  |  |  |  |
| Wergeland et al., 2014 | 182 | 11.5 | 8-15 | 1. Individual CBT | FRIENDS |  | 10 | Wergeland et al., 2014 | Primary diagnosis (SAD) | Primary diagnosis (GAD, SoAD) |
|  |  |  |  | 2. Group CBT | FRIENDS |  |  | Bjaastad et al., 2023 | Therapist competence | Therapist adherence, Therapeutic alliance |
|  |  |  |  | 3. Waitlist | - |  |  |  |  |  |
| Wood et al., 2006 | 35 | 10.0 | 6-13 | 1. Family CBT | Building Confidence |  | 12-16 | Wood et al., 2009 |  | Age |
|  |  |  |  | 2. Individual CBT | Coping CAT |  |  |  |  |  |
| Wright et al., 2022 | 268 | 9.6 | 7-16 | 1. Individual CBT | OST |  | 1 | Wright et al., 2022 | Stimulus, Treatment preferences | Phobia Diagnosis (type) |
|  |  |  |  | 2. Individual CBT | Standard/Manual |  | 6-12 |  | Age, phobia severity, |  |
| Barrett et al., 1996 | 79 | 9.3 | 7-14 | 1. Child CBT | Coping Koala | Modified or Augmented CBT | 12 | Barrett et al., 1996 | Primary diagnosis (GAD, SAD, SoAD) | Age, Sex |
|  |  |  |  | 2. Child CBT | Coping Koala |  |  |  |  |  |
|  |  |  |  |  | + FAM (Coping Cat adaptation) |  |  |  |  |  |
|  |  |  |  | 3. Waitlist | - |  |  |  |  |  |
|  |  |  |  |  |  |  |  |  |  |  |
| Nauta et al., 2003 | 79 | 11.0 | 7-18 | 1. Child CBT | Coping CAT (Dutch) |  | 12 | Nauta et al., 2003 | Age, Sex |  |
|  |  |  |  | 2. Child CBT | Coping CAT (Dutch) |  |  |  | Duration of anxiety |  |
|  |  |  |  |  | + CPT (7 sessions) |  |  |  |  |  |
|  |  |  |  | 3. Waitlist | - |  |  |  |  |  |
| Pina et al., 2012 | 88 | 10.4 | - | 1. Child CBT | Manual/ Standard |  | 12 | Pina et al., 2012 | Ethnicity |  |
|  |  |  |  | 2. Family CBT | Dyadic parent groups |  |  |  | Language |  |
| Silverman et al., 2022 | 341 | 10 | 7-16 | 1. Child CBT | Manual/ Standard |  |  | Silverman et al., 2023 | Parent positive reinforcement | Parent negative reinforcement |
|  |  |  |  | 2. Child CBT | Manual/ Standard |  | 12-14 |  | Parent psychological control | Parental acceptance |
|  |  |  |  |  | + PRTa (Reinforcement) |  |  | Patriarca et al., 2022 | Parental Enculturation | Parental Acculturation |
|  |  |  |  | 3. Child CBT | Manual/ Standard |  |  |  | Country of origin |  |
|  |  |  |  |  | + PRTb (Relationship) |  |  |  |  |  |
| Rapee et al., 2017 | 281 | 9.3 | 6-16 | 1. Individual CBT | Cool Kids |  | 10 | Rapee et al., 2020 | Age, Sex |  |
|  |  |  |  | 2. Child CBT | Parent Bibliotherapy (6-13 yrs) |  |  |  | Baseline anxiety, Comorbid mood disorder |  |
|  |  |  |  | 3. Child CBT | Cool Teens (>13 yrs) |  |  |  | Parent education |  |
| Rapee et al., 2023 | 200 | 9.5 | 7-16 | 1. Child CBT | Cool Kids |  | 10 | Rapee et al., 2023 | Age, Sex, |  |
|  |  |  |  | 2. Child CBT | Cool Kids - Social Anxiety |  |  |  | Primary diagnosis (SAD) |  |
| Suveg et al., 2018 | 92 | 9.0 | 7-12 | 1. Child CBT | ECBT |  | 10 | Suveg et al., 2018 | Child emotion dysregulation |  |
|  |  |  |  | 2. Child CBT | Coping CAT |  |  |  |  |  |
| Thirlwall et al., 2013 | 194 | 10.0 | 7-12 | 1. Child CBT | Full Guided PCBT |  | 8 | Thirlwall et al., 2013 | Therapist training/experience |  |
|  |  |  |  | 2. Child CBT | Brief Guided PCBT |  | 4 |  |  |  |
|  |  |  |  | 3. Waitlist | - |  |  |  |  |  |
| Waters et al., 2013 | 37 | 9.6 | 7-13 | 1. Group CBT | ABMT - ATP |  | 12 | Waters et al., 2013 | Attention bias |  |
|  |  |  |  | 2. Child CBT | ABMT - ATC |  |  |  |  |  |
| Wood et al., 2020 | 167 | 9.9 | 7-13 | 1. Child CBT | Coping CAT |  | 16 | Storch et al., 2021 | IQ | Age |
|  |  |  |  | 2. Child CBT | BIACA |  |  |  | Externalising subscale | Internalising subscale |
|  |  |  |  | 3. TAU | Active standard care |  |  |  |  | Total problems subscale |
|  |  |  |  |  |  | Modified or Augmented CBT |  |  |  | Autism symptoms |
|  |  |  |  |  |  |  |  | Hollocks et al., 2022 | Social cognition | Decision making |
|  |  |  |  |  |  |  |  | Guzick et al., 2024 | Age |  |
| deLijster et al., 2019 | 55 | 11.2 | 8-16 | 1. Child CBT | FRIENDS for Life (Dutch) |  | 10 | deLijster et al., 2019 | Age |  |
|  |  |  |  |  | + online ABM |  | 9 |  | Attention bias |  |
|  |  |  |  | 2. Child CBT | FRIENDS for Life (Dutch) |  | 10 |  |  |  |
|  |  |  |  |  | + online ACC |  | 9 |  |  |  |
| Liber et al., 2008 | 127 | 10.0 | 8-12 | 1. Child CBT | FRIENDS for Life (Dutch) |  | 10 | Liber et al., 2008 |  | Primary diagnosis (SoAD) |
|  |  |  |  |  | + Parent psychoeducation |  | 4 | Liber et al., 2010 | Treatment adherence | Therapeutic alliance |
|  |  |  |  | 2. Group CBT | FRIENDS for Life (Dutch) |  |  |  |  |  |
|  |  |  |  |  | + Parent psychoeducation |  |  |  |  |  |
| Manassis et al., 2002 | 78 | 10.0 | 8-12 | 1. Individual CBT | Coping CAT |  | 12 | Manassis et al., 2002 | Sex | Social Anxiety Symptoms |
|  |  |  |  |  | + Parent psychoeducation |  |  |  | Hyperactivity level |  |
|  |  |  |  | 2. Group CBT | Coping Bear |  | 12 |  |  |  |
|  |  |  |  |  | + Parent psychoeducation |  |  |  |  |  |
| de Jong et al., 2023 | 55 | 9.3 | 8-12 | 1. Child CBT | Therapist-guided exposure |  | 3 | de Jong et al., 2023 | Parental modelling/reassurance | Parental anxiety |
|  |  |  |  | 2. Child CBT | Minimally guided exposure |  |  |  | Parental avoidance |  |
|  |  |  |  | 3. Child CBT | Parent-guided exposure |  |  |  | Parental reinforcement |  |
| Farrell et al., 2018 | 35 | 10.4 | 7-14 | 1. Child CBT | OST |  | 1 | Farrell et al., 2018 | Within-session fear reduction | Age |
|  |  |  |  |  | + Pill Placebo |  |  |  |  |  |
|  |  |  |  | 2. Child CBT | OST |  |  |  |  |  |
|  |  |  |  |  | + Medication: DCS |  |  |  |  |  |
| Ginsburg et al., 2021 | 54 | 8.0 | 5-12 | 1. Child CBT | CALM |  | 6 | Caron et al., 2020 | Therapist adherence (Nurse) | Therapist self-efficacy (Nurse) |
|  |  |  |  | 2. Child CBT | CALM-R (Relaxation only) |  |  |  |  |  |
| Ollendick et al., 2015 | 97 | 8.9 | 6-15 | 1. Child CBT | OST |  | 1 | Ollendick et al., 2015 | Age, Sex |  |
|  |  |  |  | 2. Child CBT | OST |  |  |  | Internalising / Externalising problems |  |
|  |  |  |  |  | + Parent Psychoeducation |  |  |  | Parental anxiety, Parental overprotection |  |
| Rapee et al., 2016 | 51 | 9.2 | 7-14 | 1. Child CBT | Module: Exposure |  | 4 | Rapee et al., 2016 | Age |  |
|  |  |  |  |  | + Medication: DCS |  |  |  | Exposure quality |  |
|  |  |  |  | 2. Child CBT | Module: Exposure |  |  |  |  |  |
|  |  |  |  |  | + Pill Placebo |  |  |  |  |  |
| Walkup et al., 2008 | 488 | 10.7 | 7-17 | 1. Child CBT | Coping CAT | Combination of controls | 14 | Albano et al., 2018 | Age, Primary diagnosis |  |
|  |  |  |  | 2. Medication | Sertraline (SRT) |  |  | Chiappini et al., 2020 | Youth involvement |  |
|  |  |  |  |  |  |  |  |  | Therapist involvement |  |
|  |  |  |  | 3. Medication | Pill Placebo |  |  | Compton et al., 2014 | Age, Sex, Ethnicity | Primary Diagnosis (SAD) |
|  |  |  |  | 4. COMB | CBT+SRT combination |  |  |  | SES, Referral source, | Primary Diagnosis (SoAD) |
|  |  |  |  |  |  |  |  |  | Baseline anxiety, Externalising disorder | Primary Diagnosis (GAD) |
|  |  |  |  |  |  |  |  |  | Parent psychopathology, Parental negativity/stress |  |
|  |  |  |  |  |  |  |  |  | Family functioning, Caregiver strain |  |
|  |  |  |  |  |  |  |  |  | Treatment expectancy |  |
|  |  |  |  |  |  |  |  | Gonzalez et al., 2015 |  | Parental anxiety |
|  |  |  |  |  |  |  |  | Halldorsdottir et al., 2015 | ODD diagnosis | ADHD diagnosis |
|  |  |  |  |  |  |  |  | Makover et al., 2020 | Age |  |
|  |  |  |  |  |  |  |  | Norris et al., 2019 | Age, Treatment expectancy |  |
|  |  |  |  |  |  |  |  | Norris et al., 2020 | Latent Class Profiles |  |
|  |  |  |  |  |  |  |  | Sanchez et al., 2019 | Age | Sex |
|  |  |  |  |  |  |  |  | Taylor et al., 2018 | Age, Sex, SES, IQ | Ethnicity |
|  |  |  |  |  |  |  |  |  | Family burden, Family functioning | Depressive symptoms |
|  |  |  |  |  |  |  |  |  | Primary diagnosis (GAD, SAD, SoAD) | Parent psychopathology |
|  |  |  |  |  |  |  |  |  | Parental treatment expectancy | Primary diagnosis (SM) |
|  |  |  |  |  |  |  |  |  | Child bias to threat | Panic subscale |
|  |  |  |  |  |  |  |  |  | Child's global functioning | Separation anxiety subscale |
|  |  |  |  |  |  |  |  |  |  | School phobia subscale |
|  |  |  |  |  |  |  |  |  |  | Thought problem subscale |
|  |  |  |  |  |  |  |  |  |  | Rule-breaking subscale |
|  |  |  |  |  |  |  |  |  |  | Child coping ability |
| *Notes:* ^a^ Intervention and control conditions: | *CBT: Cognitive Behavioural Therapy (Individual child, Group, or Family); TAU: Treatment as Usual; COMB: Combination treatment (i.e., CBT+medication).* | | | | | | | | | |
| ^b^ Program description: | Standard CBT programs (10-16 sessions): CBGT-A: Cognitive Behavioural Group Therapy for Adolescents; Coping CAT; Coping Koala; Coping Bear; Cook Kids; FRIENDS; Building Confidence | | | | | | | | | |
|  | Variations of standard CBT: SET-C: Social Effectiveness Therapy for Children; ABMT: Attention Bias Modification Treatment; BBT: Brief Behavioural Therapy; M-CBT: modular CBT; I-CBT: Internet CBT; ISUPPORT: Internet-delivered supportive therapy; Camp-Cope-A-Lot; BIACA: Behavioural Interventions for Anxiety in Children with Autism; ECBT: Emotion-focused CBT; Parent Bibliotherapy; Cool Teens CD-ROM. | | | | | | | | | |
|  | Modified CBT includes altered sessions or content (average 1-8 sessions): SSL: Super Skills for Life; AIP: Adolescent Intensive Panic Treatment; OST: One Session Treatment; CALM: Child Anxiety Learning Modules and CALM-R (relaxation module only); Specific Modules (exposure only); Full and Brief Parent CBT; ATP: Attention towards Positive Stimuli. | | | | | | | | | |
|  | Augmentation refers to additional modules: PI: Parental involvement; Online ABM: Attention Bias Modification; online ACC: Attention Control; CPT: Cognitive Parent Training; Parent Psychoeducation modules; Sleeping Tigers; FAM: Family Management or Parental Involvement; PRTa: Parent Reinforcement Training; PRTb: Parent Relationship Training | | | | | | | | | |
|  | Medication: Pill placebo, DCS: D-cycloserine; SRT: Sertraline | | | | | | | | | |
|  | TAU: Testbusters; ES: Educational support; ACT: Attention Control Training; ST: Supportive Therapy; FESA: Family Education/Support/Attention; ARC: Assisted Referral to Care; TAU variety* refers to Australian CAMHS services; Active standard care: standard/maintained therapy or medication; play therapy, family therapy, supportive psychotherapy, non-CBT approaches. | | | | | | | | | |
|  | Alternative psychotherapy refers to other active therapeutic services: SPACE: Supportive Parenting for Anxious Childhood Emotions; CCT: Child Centered Therapy. | | | | | | | | | |
| ^c^ Moderator variables: | GAD: generalised anxiety disorder; SAD: separation anxiety disorder; SP: specific phobia; SoAD: social anxiety disorder; AD: anxiety disorder; EMA: ecological momentary assessment; ADHD: attention deficit/hyperactivity disorder; IQ: intelligence quotient; SRT: medication sertraline; SES: socio-economic status; ODD: oppositional defiance disorder; SM: selective mutism. | | | | | | | | | |

**D. RISK OF BIAS ASSESSMENTS FOR RANDOMISED CONTROLLED TRIALS**

| **Trial** | **Author** | **Randomisation Process** | **Deviation from intended Intervention** | **Missing Outcome Data** | **Measure of Outcome** | **Selection of Reported Results** |
| --- | --- | --- | --- | --- | --- | --- |
| 1 | Barrett et al., 1996 | S | S | S | S | S |
| 2 | Barrington et al., 2005 | S | S | S | S | S |
| 3 | Beidel et al., 2000 | L | S | S | S | S |
| 4 | Bodden et al., 2008 | S | L | L | S | L |
| 5 | deJong et al., 2023 | L | L | L | S | L |
| 6 | deLijster et al., 2019 | L | S | L | L | L |
| 7 | Diego et al., 2024 | L | S | L | S | L |
| 8 | Farrell et al., 2018 | S | S | L | L | L |
| 9 | Ginsburg et al., 2019 | S | L | L | S | L |
| 10 | Ginsburg et al., 2020 | S | L | L | S | L |
| 11 | Hayward et al., 2000 | S | S | L | S | S |
| 12 | Kendall et al., 1997 | S | L | S | S | S |
| 13 | Kendall et al., 2008 | S | L | L | S | L |
| 14 | Lebowitz et al., 2019 | S | L | L | S | L |
| 15 | Liber et al., 2008 | S | L | L | S | L |
| 16 | Manassis et al., 2002 | S | L | S | S | S |
| 17 | Nauta et al., 2003 | S | S | L | S | L |
| 18 | Nordh et al., 2021 | L | S | L | S | L |
| 19 | Ollendick et al., 2009 | L | S | L | S | S |
| 20 | Ollendick et al., 2015 | S | S | L | S | L |
| 21 | Ollendick et al., 2019 | L | S | L | S | L |
| 22 | Pina et al., 2012 | S | S | S | S | S |
| 23 | Pincus 2015* | S | S | L | S | L |
| 24 | Rapee et al., 2016 | L | L | L | L | S |
| 25 | Rapee et al., 2017 | L | S | L | S | L |
| 26 | Rapee et al., 2023 | L | S | L | S | L |
| 27 | Silk et al., 2016 | S | S | L | S | L |
| 28 | Silverman et al., 1999 | S | L | L | S | S |
| 29 | Silverman et al., 2019 | S | L | L | S | L |
| 30 | Silverman et al., 2022 | L | L | L | S | L |
| 31 | Storch et al., 2013 | S | S | L | S | L |
| 32 | Storch et al., 2015a | S | S | L | S | L |
| 33 | Storch et al., 2015b | S | L | L | S | L |
| 34 | Suveg et al., 2018 | L | S | L | S | S |
| 35 | Thirlwall et al., 2013 | S | S | L | S | L |
| 36 | Villabö et al., 2018 | S | L | L | S | L |
| 37 | Walkup et al., 2008 | L | S | L | S | L |
| 38 | Weersing et al., 2017 | S | L | L | L | L |
| 39 | Wergeland et al., 2014 | S | L | L | S | L |
| 40 | Wood et al., 2006 | L | L | L | S | L |
| 41 | Wood et al., 2015 | L | L | L | S | L |
| 42 | Wood et al., 2020 | S | S | L | S | L |
| 43 | Wright et al., 2022 | L | L | L | S | L |
| 44 | Waters et al., 2013 | L | L | L | L | L |
| *Note:* | | | | | | |

**E. MODERATOR QUALITY ANALYSIS**

| **Study** | **Author** | **Type** | **a Priori hypothesis (1a)** | **a Priori power (1b)** | **Theory/ evidence driven (2)** | **Measured pre-randomisation (3)** | **Quality of measurement (4)** | **Explicit interaction**  **test (5)** | **Type of evidence** |
| --- | --- | --- | --- | --- | --- | --- | --- | --- | --- |
| 1 | Diego et al., 2024 | O | + | N/A | + | + | + | + | Exploratory |
| 2 | Hayward et al., 2000 | O | - | N/A | + | + | + | - | Insufficient |
| 3 | Kendall et al., 1997 | O | + | N/A | + | + | + | + | Confirmatory |
| 4 | Silverman et al., 1999 | O | - | N/A | - | + | + | + | Exploratory |
| 5 | Beidel et al., 2000 | O | - | N/A | - | + | + | N/I | Insufficient |
| 6 | Ollendick et al., 2009 | O | - | N/A | + | + | + | - | Insufficient |
| 7 | Halldorsdottir et al., 2016 | S | N/A | - | + | + | + | + | Exploratory |
| 8 | Ollendick et al., 2019 | O | - | N/A | + | + | + | + | Exploratory |
| 9 | Weersing et al., 2017 | O | + | N/A | + | + | + | + | Confirmatory |
| 10 | Brent et al., 2020 | S | N/A | + | + | + | + | + | Confirmatory |
| 11 | Schwartz et al., 2023 | S | N/A | + | + | + | + | + | Confirmatory |
| 12 | Barrington et al., 2005 | O | + | N/A | + | + | + | N/I | Insufficient |
| 13 | Ginsburg et al., 2020 | O | + | N/A | + | + | + | + | Confirmatory |
| 14 | Nordh et al., 2022 | S | N/A | - | + | + | + | + | Exploratory |
| 15 | Smarason et al., 2023 | S | N/A | - | + | + | + | + | Exploratory |
| 16 | Kim et al., 2019 | S | N/A | - | + | + | + | + | Exploratory |
| 17 | Silk et al., 2016 | O | + | N/A | + | + | + | + | Confirmatory |
| 18 | Wallace et al., 2017 | S | N/A | - | + | + | + | + | Exploratory |
| 19 | Silk et al., 2019 | S | N/A | - | + | + / - | + | + | Exploratory (baseline) Insufficient (post) |
| 20 | Lebowitz et al., 2021 | S | N/A | - | + | + / - | + | + | Exploratory (baseline) Insufficient (post) |
| 21 | Zilcha-Mano et al., 2020 | S | N/A | - | + | + / - | + | + | Exploratory (baseline) Insufficient (post) |
| 22 | Zilcha-Mano et al., 2023 | S | N/A | - | + | + / - | + | + | Exploratory (baseline) Insufficient (post) |
| 23 | Bodden et al., 2008 | O | + | N/A | + | + | + | + | Confirmatory |
| 24 | Maric et al., 2018 | S | N/A | - | + | + | + | + | Exploratory |
| 25 | Kendall et al., 2008 | O | + | N/A | + | + | + | + | Confirmatory |
| 26 | Suveg et al., 2009 | S | N/A | - | + | + | + | + | Exploratory |
| 27 | Puleo et al., 2011 | S | N/A | - | + | + | + | + | Exploratory |
| 28 | Hardway et al., 2015 | S | N/A | - | + | + | + | + | Exploratory |
| 29 | Elkins et al., 2016 | S | N/A | - | + | + | + | + | Exploratory |
| 30 | Vaclavik et al., 2017 | S | N/A | - | + | + | + | + | Exploratory |
| 31 | Villabö et al., 2018 | O | + | N/A | + | + | + | + | Confirmatory |
| 32 | Helland et al., 2022 | S | N/A | - | + | + | + | + | Exploratory |
| 33 | Wergeland et al., 2014 | O | + | N/A | + | + | + | + | Confirmatory |
| 34 | Bjaastad et al., 2023 | S | N/A | - | + | + / - | + | + | Exploratory (baseline) Insufficient (post) |
| 35 | Wood et al., 2009 | S | N/A | - | + | + | + | + | Exploratory |
| 36 | Wright et al., 2022 | O | + | N/A | + | + | + | + | Confirmatory |
| 37 | Barrett et al., 1996 | O | + | N/A | + | + | + | + | Confirmatory |
| 38 | Nauta et al., 2003 | O | + | N/A | + | + | + | + | Confirmatory |
| 39 | Pina et al., 2012 | O | + | N/A | + | + | + | + | Confirmatory |
| 40 | Silverman et al., 2023 | S | N/A | + | + | + | + | + | Confirmatory |
| 41 | Patriarca et al., 2022 | S | N/A | - | + | + | + | + | Confirmatory |
| 42 | Rapee et al., 2020 | O | + | N/A | + | + | + | + | Confirmatory |
| 43 | Rapee et al., 2023 | O | - | N/A | + | + | + | + | Confirmatory |
| 44 | Suveg et al., 2018 | O | + | N/A | + | + | + | + | Confirmatory |
| 45 | Thirlwall et al., 2013 | O | + | N/A | + | + | + | + | Confirmatory |
| 46 | Waters et al., 2013 | O | + | N/A | + | + | + | + | Confirmatory |
| 47 | Storch et al., 2021 | S | N/A | + | + | + | + | + | Confirmatory |
| 48 | Hollocks et al., 2022 | S | N/A | - | + | + | + | + | Exploratory |
| 49 | Guzick et al, 2024 | S | N/A | - | + | + | + | + | Exploratory |
| 50 | deLijster et al., 2019 | O | + | N/A | + | + | + | + | Confirmatory |
| 51 | Liber et al., 2008 | O | - | N/A | + | + | + | + | Exploratory |
| 52 | Liber et al., 2010 | S | N/A | + | + | - | + | + | Insufficient |
| 53 | Manassis et al., 2002 | O | + | N/A | + | + | + | + | Confirmatory |
| 54 | de Jong et al., 2023 | O | + | N/A | + | + | + | + | Confirmatory |
| 55 | Farrell et al., 2018 | O | + | N/A | + | + / - | + | + | Exploratory (baseline) Insufficient (post) |
| 56 | Caron et al., 2020 | S | N/A | - | + | - | - | + | Insufficient |
| 57 | Ollendick et al., 2015 | O | + | N/A | + | + | + | + | Confirmatory |
| 58 | Rapee et al., 2016 | O | + | N/A | + | - | - | + | Insufficient |
| 59 | Albano et al., 2018 | S | N/A | - | + | + | + | + | Exploratory |
| 60 | Chiappini et al., 2020 | S | N/A | - | + | - | + | + | Insufficient |
| 61 | Compton et al., 2014 | S | N/A | - | - | + | + | + | Exploratory |
| 62 | Gonzalez et al., 2015 | S | N/A | - | + | + | + | + | Exploratory |
| 63 | Halldorsdottir et al., 2015 | S | N/A | - | + | + | + | + | Exploratory |
| 64 | Makover et al., 2020 | S | N/A | - | + | + | + | + | Exploratory |
| 65 | Norris et al., 2019 | S | N/A | - | + | - | - | + | Insufficient |
| 66 | Norris et al., 2020 | S | N/A | - | + | + | + | + | Exploratory |
| 67 | Sanchez et al., 2019 | S | N/A | - | + | + | + | + | Exploratory |
| 68 | Taylor et al., 2018 | S | N/A | - | - | + | + | + | Exploratory |
|  |  |  |  |  |  |  |  |  |  |

**F. VARIABLES EXAMINED AND SIGNIFICANTLY ASSOCIATED WITH DIFFERENTIAL TREATMENT EFFECTS**

|  | |  | **Inactive** | | | **Active non-CBT** | | | | **Active CBT** | | | | **Total** | | | | | |  |
| --- | --- | --- | --- | --- | --- | --- | --- | --- | --- | --- | --- | --- | --- | --- | --- | --- | --- | --- | --- | --- |
| **Moderator variables** | | **Time** | **Examined** | **Associated** | | **Examined** | | **Associated** | | **Examined** | | **Associated** | | **Examined** | | **Associated** | | **Irrelevant** | |  |
| **Demographic Variables** | | | | | | | | | | | | | | | | | | | | |
| 1 | Age | Invariant | 0 | | 4 | | 2 | | 7 | | 5 | | 21 | | 32 | | 7 | | 25 | |
| 2 | Sex | Invariant | 0 | | 4 | | 0 | | 6 | | 3 | | 10 | | 20 | | 3 | | 17 | |
| 3 | Pubertal status | Invariant | 0 | | 0 | | 0 | | 1 | | 0 | | 0 | | 1 | | 0 | | 1 | |
| 4 | Ethnicity/Race | Invariant | 3 | | 5 | | 0 | | 1 | | 1 | | 3 | | 9 | | 4 | | 5 | |
| 5 | Socioeconomic Status (SES) | Variant | 0 | | 0 | | 0 | | 0 | | 0 | | 2 | | 2 | | 0 | | 2 | |
| 6 | IQ | Variant | 0 | | 0 | | 0 | | 0 | | 0 | | 2 | | 2 | | 0 | | 2 | |
| 7 | Language | Invariant | 0 | | 0 | | 0 | | 0 | | 0 | | 1 | | 1 | | 0 | | 1 | |
| **Contextual / Familial Variables** | | | | | | | | | | | | | | | | | | | | |
| 8 | Parental psychopathology (anxiety/depression) | Variant | 0 | | 0 | | 0 | | 3 | | 5 | | 7 | | 10 | | 5 | | 5 | |
| 9 | Parental stress/negativity | Variant | 0 | | 0 | | 1 | | 1 | | 0 | | 1 | | 2 | | 1 | | 1 | |
| 10 | Family accommodation | Variant | 0 | | 0 | | 1 | | 3 | | 0 | | 0 | | 3 | | 1 | | 2 | |
| 11 | Parental acculturation | Variant | 0 | | 0 | | 0 | | 0 | | 2 | | 2 | | 2 | | 2 | | 0 | |
| 12 | Parental enculturation | Variant | 0 | | 0 | | 0 | | 0 | | 0 | | 1 | | 1 | | 0 | | 1 | |
| 13 | Parental identification with  the country of origin | Variant | 0 | | 0 | | 0 | | 0 | | 0 | | 2 | | 2 | | 0 | | 2 | |
| 14 | Parental education | Variant | 0 | | 0 | | 1 | | 1 | | 0 | | 1 | | 2 | | 1 | | 1 | |
| 15 | Family functioning | Variant | 0 | | 0 | | 0 | | 0 | | 0 | | 2 | | 2 | | 0 | | 2 | |
| 16 | Family burden/caregiver strain | Variant | 0 | | 0 | | 0 | | 1 | | 0 | | 2 | | 3 | | 0 | | 3 | |
| 17 | Parental overprotectiveness | Variant | 0 | | 0 | | 0 | | 0 | | 0 | | 1 | | 1 | | 0 | | 1 | |
| 18 | Maternal / Parental acceptance | Variant | 0 | | 0 | | 0 | | 1 | | 1 | | 1 | | 2 | | 1 | | 1 | |
| 19 | Maternal / Parental Psychological Control | Variant | 0 | | 0 | | 0 | | 1 | | 0 | | 1 | | 2 | | 0 | | 2 | |
| 20 | Parental reinforcement | Variant | 0 | | 0 | | 0 | | 0 | | 1 | | 2 | | 2 | | 1 | | 1 | |
| 21 | Parental avoidance | Variant | 0 | | 0 | | 0 | | 0 | | 0 | | 1 | | 1 | | 0 | | 1 | |
| 22 | Parental modelling/reassurance | Variant | 0 | | 0 | | 0 | | 0 | | 0 | | 1 | | 1 | | 0 | | 1 | |
| **Clinical Variables** | | | | | | | | | | | | | | | | | | | | |
| 23 | Primary anxiety diagnosis | Variant | 0 | | 1 | | 0 | | 4 | | 6 | | 9 | | 14 | | 6 | | 8 | |
| 24 | Baseline anxiety (severity) | Variant | 1 | | 1 | | 3 | | 5 | | 0 | | 3 | | 9 | | 4 | | 5 | |
| 25 | Anxiety (duration/age of onset) | Variant | 0 | | 0 | | 0 | | 1 | | 0 | | 1 | | 2 | | 0 | | 2 | |
| 26 | Number of anxiety disorders | Variant | 0 | | 0 | | 1 | | 1 | | 0 | | 0 | | 1 | | 1 | | 0 | |
| 27 | Comorbid symptoms - anxiety | Variant |  | |  | |  | |  | |  | |  | |  | |  | |  | |
|  | *Separation scale/panic scores/school phobia* |  |  | |  | |  | |  | | 1 | | 1 | | 1 | | 1 | | 0 | |
|  | *Fear and avoidance scales* |  |  | |  | |  | |  | | 1 | | 1 | | 1 | | 1 | | 0 | |
|  | *Social symptoms* |  |  | |  | |  | |  | | 1 | | 1 | | 1 | | 1 | | 0 | |
| 28 | Comorbid symptoms - internalising (depressive) | Variant |  | |  | |  | |  | |  | |  | |  | |  | |  | |
|  | - internalising subscale |  | 0 | | 0 | | 0 | | 1 | | 1 | | 2 | | 3 | | 1 | | 2 | |
|  | - depressive symptoms |  | 2 | | 3 | | 1 | | 2 | | 1 | | 1 | | 6 | | 4 | | 2 | |
| 29 | Comorbid symptoms - externalising (ADHD) | Variant |  | |  | |  | |  | |  | |  | |  | |  | |  | |
|  | - *Thought problem scale / Rule-breaking scale* |  | 0 | | 0 | | 0 | | 0 | | 1 | | 2 | | 2 | | 1 | | 1 | |
|  | - *ADHD symptoms* |  | 0 | | 1 | | 0 | | 0 | | 2 | | 2 | | 3 | | 2 | | 1 | |
|  | - *Externalising subscale* |  | 0 | | 0 | | 0 | | 2 | | 0 | | 3 | | 5 | | 0 | | 5 | |
| 30 | Comorbid symptoms - autism | Variant | 0 | | 0 | | 0 | | 0 | | 2 | | 2 | | 2 | | 2 | | 0 | |
| 31 | Comorbid status (additional disorders, yes/no) | Variant | 0 | | 4 | | 0 | | 1 | | 1 | | 3 | | 8 | | 1 | | 7 | |
| 32 | Global functioning | Variant | 0 | | 0 | | 0 | | 1 | | 0 | | 1 | | 2 | | 0 | | 2 | |
| **Treatment Variables** | | | | | | | | | | | | | | | | | | | | |
| 33 | Involvement (parent, child, therapist) | Variant | 0 | | 0 | | 0 | | 1 | | 0 | | 2 | | 3 | | 0 | | 3 | |
| 34 | Referral Source | Invariant | 0 | | 0 | | 0 | | 0 | | 0 | | 1 | | 1 | | 0 | | 1 | |
| 35 | Treatment site/country | Invariant | 0 | | 2 | | 0 | | 0 | | 0 | | 0 | | 2 | | 0 | | 2 | |
| 36 | Exposure quality | Variant | 0 | | 0 | | 0 | | 0 | | 0 | | 1 | | 1 | | 0 | | 1 | |
| 37 | Within-session fear reduction | Variant | 0 | | 0 | | 0 | | 0 | | 0 | | 1 | | 1 | | 0 | | 1 | |
| 38 | Stimulus | Variant | 0 | | 0 | | 0 | | 0 | | 0 | | 1 | | 1 | | 0 | | 1 | |
| 39 | Treatment preference | Variant | 0 | | 0 | | 0 | | 0 | | 0 | | 1 | | 1 | | 0 | | 1 | |
| 40 | Treatment expectancy (parent/child/therapist) | Variant | 0 | | 0 | | 0 | | 0 | | 0 | | 3 | | 3 | | 0 | | 3 | |
| 41 | Therapeutic alliance | Variant | 0 | | 0 | | 0 | | 0 | | 1 | | 1 | | 1 | | 1 | | 0 | |
| 42 | Treatment response | Variant | 0 | | 0 | | 0 | | 2 | | 0 | | 0 | | 2 | | 0 | | 2 | |
| 43 | Previous treatment received | Invariant | 0 | | 0 | | 0 | | 1 | | 0 | | 0 | | 1 | | 0 | | 1 | |
| 44 | Therapist Adherence | Variant | 0 | | 0 | | 0 | | 0 | | 1 | | 3 | | 3 | | 1 | | 2 | |
| 45 | Therapist Efficacy | Variant | 0 | | 0 | | 0 | | 0 | | 1 | | 1 | | 1 | | 1 | | 0 | |
| 46 | Therapist training/experience/  competence | Variant | 0 | | 0 | | 0 | | 0 | | 0 | | 2 | | 2 | | 0 | | 2 | |
| 47 | Therapist pairing | Invariant | 0 | | 1 | | 0 | | 0 | | 0 | | 0 | | 1 | | 0 | | 1 | |
| **Child variables and other factors** | | | | | | | | | | | | | | | | | | | | |
| 48 | Coping ability | Variant | 0 | | 0 | | 0 | | 0 | | 1 | | 1 | | 1 | | 1 | | 0 | |
| 49 | Decision-making | Variant | 0 | | 0 | | 0 | | 0 | | 1 | | 1 | | 1 | | 1 | | 0 | |
| 50 | Social cognition | Variant | 0 | | 0 | | 0 | | 0 | | 0 | | 1 | | 1 | | 0 | | 1 | |
| 51 | Parent oxytocin levels | Variant | 0 | | 0 | | 0 | | 1 | | 0 | | 0 | | 1 | | 0 | | 1 | |
| 52 | Child oxytocin levels | Variant | 0 | | 0 | | 2 | | 2 | | 0 | | 0 | | 2 | | 2 | | 0 | |
| 53 | EMA sleep variables | Variant | 0 | | 0 | | 1 | | 1 | | 0 | | 0 | | 1 | | 1 | | 0 | |
| 54 | Interpersonal negative events | Variant | 0 | | 0 | | 1 | | 1 | | 0 | | 0 | | 1 | | 1 | | 0 | |
| 55 | Attention bias (bias to threat) | Variant | 0 | | 1 | | 0 | | 0 | | 0 | | 3 | | 4 | | 0 | | 4 | |
| 56 | Attention control | Variant | 1 | | 1 | | 0 | | 0 | | 0 | | 0 | | 1 | | 1 | | 0 | |
| 57 | Social skills | Variant | 0 | | 0 | | 0 | | 1 | | 0 | | 0 | | 1 | | 0 | | 1 | |
| 58 | Emotion regulation/dysregulation | Variant | 0 | | 0 | | 0 | | 0 | | 1 | | 2 | | 2 | | 1 | | 1 | |
| 59 | Latent Class Profiles | Invariant | 0 | | 0 | | 0 | | 0 | | 0 | | 1 | | 1 | | 0 | | 1 | |
| 60 | Anticipatory anxiety | Variant | 0 | | 0 | | 0 | | 1 | | 0 | | 0 | | 1 | | 0 | | 1 | |
| 61 | Post-event processing | Variant | 0 | | 0 | | 0 | | 1 | | 0 | | 0 | | 1 | | 0 | | 1 | |
| 62 | Internal/External focus of attention | Variant | 0 | | 0 | | 0 | | 1 | | 0 | | 0 | | 1 | | 0 | | 1 | |
| 63 | Safety behaviours | Variant | 0 | | 0 | | 0 | | 1 | | 0 | | 0 | | 1 | | 0 | | 1 | |
|  | | | | | | | | | | | | | | | | | | | | |
